# On the Operational Aspects of Measuring Nanoparticle Sizes

**DOI:** 10.3390/nano9010018

**Published:** 2018-12-23

**Authors:** Jean-Marie Teulon, Christian Godon, Louis Chantalat, Christine Moriscot, Julien Cambedouzou, Michael Odorico, Johann Ravaux, Renaud Podor, Adèle Gerdil, Aurélie Habert, Nathalie Herlin-Boime, Shu-wen W. Chen, Jean-Luc Pellequer

**Affiliations:** 1 Univ. Grenoble Alpes, CEA, CNRS, IBS, F-38000 Grenoble, France; jean-marie.teulon@cea.fr (J.-M.T.); christine.moriscot@ibs.fr (C.M.); 2 CEA, iBEB, LIRM, F-30207 Bagnols sur Cèze, France; christian.godon@cea.fr (C.G.); louischantalat@hotmail.fr (L.C.); michael.odorico@cea.fr (M.O.); 3 CEA, BIAM, LBDP, F-13108 Saint Paul lez Durance, France; 4 Institut de Chimie Séparative de Marcoule (ICSM), CEA, CNRS, ENSCM, Univ. Montpellier, F-30207 Marcoule, France; julien.cambedouzou@enscm.fr (J.C.); johann.ravaux@cea.fr (J.R.); renaud.podor@cea.fr (R.P.); 5 UMR3685 CEA-CNRS, NIMBE, LEDNA, CEA Saclay, F-91191 Gif sur Yvette, France; Adele.gm@outlook.fr (A.G.); aurelie.habert@cea.fr (A.H.); nathalie.herlin@cea.fr (N.H.-B.); 6 478 rue Cyprien Jullin, F-38470 Vinay, France; cmft551@yahoo.com

**Keywords:** nanoparticles, nanotoxicology, metrology, AFM, TEM, SEM, wet-STEM, SAXS, DLS

## Abstract

Nanoparticles are defined as elementary particles with a size between 1 and 100 nm for at least 50% (in number). They can be made from natural materials, or manufactured. Due to their small sizes, novel toxicological issues are raised and thus determining the accurate size of these nanoparticles is a major challenge. In this study, we performed an intercomparison experiment with the goal to measure sizes of several nanoparticles, in a first step, calibrated beads and monodispersed SiO_2_ Ludox®, and, in a second step, nanoparticles (NPs) of toxicological interest, such as Silver NM-300 K and PVP-coated Ag NPs, Titanium dioxide A12, P25(Degussa), and E171(A), using commonly available laboratory techniques such as transmission electron microscopy, scanning electron microscopy, small-angle X-ray scattering, dynamic light scattering, wet scanning transmission electron microscopy (and its dry state, STEM) and atomic force microscopy. With monomodal distributed NPs (polystyrene beads and SiO_2_ Ludox®), all tested techniques provide a global size value amplitude within 25% from each other, whereas on multimodal distributed NPs (Ag and TiO_2_) the inter-technique variation in size values reaches 300%. Our results highlight several pitfalls of NP size measurements such as operational aspects, which are unexpected consequences in the choice of experimental protocols. It reinforces the idea that averaging the NP size from different biophysical techniques (and experimental protocols) is more robust than focusing on repetitions of a single technique. Besides, when characterizing a heterogeneous NP in size, a size distribution is more informative than a simple average value. This work emphasizes the need for nanotoxicologists (and regulatory agencies) to test a large panel of different techniques before making a choice for the most appropriate technique(s)/protocol(s) to characterize a peculiar NP.

## 1. Introduction

Despite differences in international definitions on nanoparticles (NPs) especially concerning solubility, aggregation, and distribution threshold, current regulatory agencies consider that a primary nanomaterial is a nanoparticle (NP) when at least one of its dimensions is in the range 1–100 nm [1], values below the diffraction-limited resolution of conventional microscopy. In Europe, the 2011/696/EU commission recommendation states that a product containing primary particles for 50% or more of the particles in the number size distribution and one or more external dimension in the size range 1–100 nm is a nanomaterial [2]. It also states that the 50% threshold is adjustable between 1% and 50% when warranted by specific concerns. The European Joined Research Centre published two reports on the requirements on measurements for the implementation of the EU commission definition of the term nanomaterial by listing an ensemble of currently used techniques with pros and cons when measuring fine sizes of nanomaterials [3] and on the application of the commission 2011/696/EU rules [1]. In the latter, a description of difficulties encountered for strictly identifying nanomaterial due to the number threshold is provided. Growth of the usage of NPs in industry is continuous [4,5] and a comprehensive report has been made by the Royal Society, UK, with 21 recommendations on the application of nanotechnology and their impact on health and environment [6].

Based on their chemical nature, NPs are used for instance as mechanical reinforcement additives with carbon nanotubes, solar cells, paints, coatings, or sunscreen cosmetics with TiO_2_; wound dressing, air filters, or toothpastes with Ag; and chemical sensors, hydrogels, or drug delivery with nanolatex [7,8]. Uptake of NPs in humans occurs via inhalation, ingestion, or transdermal routes. Toxicology of NPs has been investigated for several decades [9] and the sub-discipline nanotoxicology was first coined in 2004 [10]. The currently identified toxicity mechanisms are membrane disruption, protein oxidation genotoxicity, reactive oxygen species formation, and interruption of energy transduction in cells [8]. Concerning NPs, the current paradigm stipulates that at the nano-size (<100 nm in diameter) physical and chemical properties are different from those of the same material in the bulk (non-nano) form [4]. Consequently, there is a growing concern that such NPs possess unexpected toxicological properties. Because smaller and smaller NPs expose more and more atoms at their surfaces, particle toxicology generally assumes that an increase in particle surface leads to an increase in toxicity [11].

Assessing the potential toxicity of NPs in biological systems starts with a complete and accurate NP characterization [12]. From a regulatory standpoint, the effect of NPs size was the most extensively studied property. Numerous studies claimed a greater toxicity for small NPs [13]. Beyond the case of one-dimensional nanomaterials such as carbon nanotubes, whose toxicity has intensively been debated due to their similarity with asbestos fibers [14], many examples can be found concerning more isotropic particles. Due to their small sizes, it is expected that NPs penetrate easily in tissues and cells. Deposition of carbon NPs (23 nm) accumulate more in asthmatic than normal patients [15]. Small Ag NPs have increased hemolytic toxicity compared to observations in presence of large agglomerates of Ag [16]. Small size of CdO NPs (22 ± 3 nm) reveals severe bacterial surface damages [17]. High bronchoalveolar inflammation was observed in a group of rats exposed to small TiO_2_ particles (20 nm in size) [9] compared with large-sized particles (250 nm). Similarly, small TiO_2_ or SiO_2_ NPs translocate through the Calu-3 monolayer with an increased translocation for the smallest and negatively charged NPs [18]. Exposure to SiO_2_ (15 and 30 nm) exerts toxic effects on HaCaT cells by altering protein expression and the cellular apoptosis is also greatly increased compared with micro SiO_2_ (365 nm) [19]. Some recent studies have found that, even at the nanoscale level, small SiO_2_ NPs (15 nm) show higher toxicity in Caco-2 cells than larger SiO_2_ NPs (55 nm) and the observed genotoxic effects are likely mediated through oxidative stress rather than a direct interaction with DNA [20]. In addition, it has been observed that SiO_2_ NPs (70 nm) penetrate the cell nucleus of human epithelial cells [21], whereas small latex NP beads (<40 nm) entered epidermal cells after transcutaneous applications on human skin [22], while only neutral, small, and positively charged polysaccharide-based NPs (<60 nm) transcytozed on brain capillaries of endothelial cells [23].

However, contradictory effects regarding NPs size have been observed in the literature. For instance, it has been observed that small Ni:Fe NPs (10 ± 3 nm) are less toxic than large particles (150 ± 50 nm) and that cytotoxicity increases when particles are coated with oleic acids [24]; and similarly for small TiO_2_ NPs (10 nm) versus large NPs (>200 nm) [25]. While 70 nm-sized SiO_2_ particles were found to enter into the cell nucleus, it was also observed for NPs of larger size (>200 nm) [21]. Distinct effects of TiO_2_ and SiO_2_ have been observed on endothelial cells depending on cell types, concentrations, and exposure time [26] and similarly for CeO_2_ (8 and 35 nm) and SiO_2_ (177 and 315 nm) NPs [27]. The size of NPs has been shown to be an important parameter for the destabilization of biological membranes but it was found that only large NPs (>100 nm) induced an increased disruption of membrane domain structures [28].

The terminology “particle size” is likely technique- and application-dependent [29]. For instance, non-microscopy techniques (such as dynamic light scattering, DLS) rely on the extraction of NP sizes using basic assumptions such as sphericity, and all techniques struggle at very low or very high NP concentrations. Microscopy-based techniques suffer from severe artifacts induced by drying mainly due to the retraction of the liquid meniscus either disrupting or promoting agglomerates. In addition, electron microscopy needs to maintain an electron dose below 1 MGy/s to avoid unwanted alterations [29]. A recent review presents advantages and disadvantages of microscopy and non-microscopy techniques to determine NPs size [29]. Likely, the oldest indirect technique to determine NPs size for powders is the Brunauer–Emmett–Teller (BET) method [30]. The most frequently used techniques for determining NP sizes are the Scanning or Transmission electron microscopies (SEM or TEM, respectively) and light scattering (e.g., dynamic light scattering, DLS). Additional techniques are also often used such as atomic force microscopy (AFM), small-angle X-ray scattering (SAXS), or nanoparticle tracking analysis (NTA). A recent technique has appeared in NPs size determination (field-flow fractionation, FFF and its related variants), but it is rather a separation technique since the NP size must be determined by additional modules such as DLS or multi-angle light scattering (MALS). Finally, standard methods such as UV-vis absorption, fluorescence, gravimetry, mass spectrometry (especially ICP-MS), and others are also used for determining NPs sizes [31]. Principles of biophysical methods used to characterize NPs have been described previously in detail [32,33].

To refine the biophysical methodologies for fine characterization of NPs size, several inter-laboratory or inter-technique comparisons have been performed [31,32,34,35,36,37,38,39,40,41,42]. These comparisons involved either several groups with the same technique, several groups with different techniques on a single type of NP, or several groups with different techniques and different types of NP. All the tested methods of size analysis are subject to a variety of pitfalls [36] and it has been concluded that the analyst should be knowledgeable and skilled in the technique employed. For instance, large variations were observed among DLS, AFM, NTA, and fluorescence correlation spectroscopy [40], where up to five times variations in NP sizes were observed between DLS and TEM [35]. It was found that the techniques most prone to artifacts were those that are the most frequently used in the literature: TEM following air drying of a sample and DLS [36]. A common conclusion from these comparisons is that the source of the irreproducible results is the heterogeneity in sample preparation [39] and proper sample deposition greatly improves the analysis of the NP sizes [41]. The agglomeration of NPs in solution is often observed and sonication only reduces it and rarely leads to separation of primary particles [35] and sometimes ultrasonication was even observed to further increase variability in NP size determination [38]. It has been suggested that the lack of standardization in the application of ultrasonic treatments across laboratories is a major cause of variability in NP suspension characteristics [43] although several groups are working on NP dispersion by studying the effect of ionic strength, pH, and particle surface chemistry [44] or various dispersion methods [45]. These studies highlight the need to substantiate characterization from manufacturers, the need to form multidisciplinary teams performing measurements as close to the biological action as possible, and the need to provide a maximum transparency in reporting size and size distributions in the literature.

As concluded by previous inter-laboratory comparison experiments, the need to better characterize NPs size remains of major importance. The inter-laboratory experiment presented in this work aimed at identifying which and when a methodology is best suited for NP size characterization. The rationale of our project was to first test each technique using calibrated nanomaterials and monodispersed solutions of NPs to evaluate potential bias of each technique toward small sized NPs. To our knowledge, this work is the first comparison that includes the wet-STEM (wSTEM) technique for NP size determination. The aim of the project was also to analyze “realistic” NPs of nanotoxicology interest (Silver and Titanium oxides [46,47]) with an “as is” principle meaning without standardized protocol. Our project relied on experts in biophysical techniques (electron and atomic force microscopies, small angle X-ray scattering, and dynamic light scattering). It was requested to perform measurements of the size of NPs using classical laboratory standard protocols and methodologies. The project had three phases: first, provide measurements and their comparison on standard nanomaterials (standard polystyrene beads); second, provide measurements on monodispersed NPs (silica, Ludox) that could be characterized by all participating techniques; and, third, provide measurements on Ag and TiO_2_ NPs, which are of major concerns in nanotoxicology. A secondary goal was to provide clear indications of which technique(s) is (are) best suited for which NPs.

## 2. Materials and Methods

### 2.1. Nanoparticles

Calibrated polystyrene nanoparticles (3000 series Nanosphere™ 20 nm size standard, batch 42376, Thermo Scientific, Fremont, CA, USA) were given as a suspension; the nominal diameter was 22 ± 2 nm (using photon correlation spectroscopy measurements according to NIST). Colloidal silica nanoparticles (Ludox) in suspension were obtained from Sigma-Aldrich (Saint Louis, MO, USA): Ludox® SM-30 (Ref 420794-1L, batch MKBL2470V), Ludox® HS-40 (Ref 420816-1L, batch BCBJ2528V), and Ludox® TM-50 (Ref 420778-1L, batch MKBP2322V). A Ludox® technical literature document from the previous manufacturer (E.I. Du Pont de Nemours, Wilmington, DE, USA) provides approximate sizes for SM-30, HS-40, and TM-50 that are ~7 nm, ~12 nm, and ~22 nm, respectively; a recent document from the current manufacturer (Grace, Columbia, MD, USA) reproduces these approximate values without providing technical details of their origin. Consequently, these sizes were not considered in our study, and the Ludox® particle sizes were considered unknown. NPs of TiO_2_ (P25, Degussa, Essen, Germany) were provided as powders with a primary particle size estimated around 21 nm [48] or 23 ± 10 nm [49]. It should be emphasized that P25 NPs used in this study were obtained several years ago from Degussa, which may not reflect the modern version of P25 from Evonik. TiO_2_ food additive E171 (batch A) are those used previously [50] while TiO_2_ A12 was synthesized in the NIMBE laboratory to avoid any uncontrolled additives. NPs of silver NM-300K were provided as suspensions by the Joint Research Center (JRC, Ispra, Italy) as a performance standard [51] and were kept in the dark at 4 °C. Using exclusively electron microscopy, it was found at JRC that NM-300K has an average size of 15 nm with 99% of particles having a size <20 nm and a second small population of NM-300K around 5 nm has also been observed [51]. Silver NPs with dispersion agent of polyvinylpyrrolidone (Ag PVP) were purchased from Sigma (758329). They are given at a size <100 nm by the manufacturer and a size of 59 ± 18 nm was previously obtained with TEM [47]. Detailed instrumentations are shown below with an indication of their geographical origin (GRE for Grenoble, MAR for Marcoule, and SAC for Saclay) since similar techniques have been used in different locations. When needed, the geographical keyword is added to the name of the technique on figures and tables.

### 2.2. AFM Measurements (GRE)

Multimode III and multimode 8 with a Nanoscope V controller (Bruker, Santa Barbara, CA, USA) were used. Tapping mode or PeakForce tapping mode were used in ambient condition (air) at room temperature (24 °C). Nanoparticles were deposited on freshly cleaved mica or highly-oriented pyrolytic graphite (Bruker, Santa Barbara, CA, USA). Cantilevers used for tapping mode were Sn-doped silicon Multi40 (MPP-22200, nominal k = 0.9 N/m, f_0_ = 54 kHz, Bruker, Santa Barbara, CA, USA), RFESP (MPP-21100, k = 3 N/m, f_0_ = 69–93 kHz, Bruker, Santa Barbara, CA, USA), and RTESP (MPP-11100, k = 20–80 N/m, f_0_ = 287–325 kHz, Bruker, Santa Barbara, CA, USA), whereas, for PeakForce tapping, silicon nitride lever with a silicon tip was used (SNL: A, k = 0.35 N/m, f_0_ = 50–80 kHz; B, k = 0.12 N/m, f_0_ = 16–28 kHz; C, k = 0.24 N/m, f_0_ = 40–75 kHz; D, k = 0.06 N/m, f_0_ = 12–24 kHz). Typical AFM imaging condition was: scan rate of 1 Hz, between 512 and 2048 pixels on 512 to 2048 lines, scan size ranging from 1 to 10 μm and optimal measurements were obtained with a scan size of 1 μm with 1024 × 1024 pixels. In tapping mode, set-point was set to the minimum necessary for imaging, whereas, in peakforce tapping mode, the automated ScanAsyst mode was used.

Image processing was performed in Gwyddion [52], stripe line removal was performed with DeStripe [53], and image enhancements were performed using home-made tools [54,55]. In brief, raw AFM images were flattened using a first-order plan fit. Further line flattening was performed using leveling tools of Gwyddion. When necessary, a mask was created using Gwyddion to select nanoparticles and to exclude those for line flattening.

For spherical nanoparticles, NP height was measured using a horizontal profile section with two different approaches: for an isolated single NP, the distance between the background and the max height of the profile was measured; for assembled NP, the distance between the max height of the first and the last NP divided by the number of NPs was measured. Thus, for a spherical or a pseudo-spherical NP, height values are attributed to its diameter.

### 2.3. TEM Measurements (GRE)

Images were taken under low dose conditions (20 e^−^/ Å^2^) with a FEI T12 electron microscope (FEI, Hillsboro, OR, USA) at 120 kV using an ORIUS SC1000 camera (Gatan, Inc., Pleasanton, CA, USA). Samples were prepared according to two protocols: TEM1, the carbon flotation technique [56,57] or, TEM2, directly deposited on carbon-coated grid (S160-4 grids, Agar Scientific Ltd, Stansted, UK). For the flotation technique, 4 μL of samples were injected to the clean side of carbon protected by a mica surface; the carbon was separated from mica by floating on a water drop; and a grid was placed on top of the carbon film, which was subsequently air-dried. The substrate carbon-coated mica was produced by evaporation of a carbon in an Emitech K950X carbon coater (QUORUM Technologie Ltd., Asford, UK). For the carbon-coated technique, samples were directly deposited on the carbon-coated grid, the excess of sample was first removed by blotting with a filter paper and then air-dried. Instrument calibration was performed during regular maintenance from the manufacturer. Raw dm3 images (4008 × 2672 px) were analyzed with Gwyddion using cross-section profiles.

### 2.4. TEM Measurements (SAC)

Images were obtained using a Philips CM12 microscope at 120 kV. Samples were prepared by different protocols depending on whether they come as a power or suspension. For powders, they were dispersed by ultrasonication in acetone or ethanol. The suspensions were used as delivered or after a step of dispersion followed by vortex agitation. In both cases, a drop was deposited on a grid, excess sample was removed with a filter paper and the sample was left for drying under air a few minutes. EM grids were “Lacey Carbon (S166-3H)” or “carbon films (S160-H)” purchased from Agar. The Lacey carbon grids were preferred in the case of agglomerated samples such as TiO_2_-P25 or TiO_2_-A12. All the images obtained from TEM were analyzed using the ImageJ software (version 1.51, NIH, Bethesda, MD, USA) [58] and the line measurement tool. Calibration was regularly checked using standard grids.

### 2.5. Wet-STEM (wSTEM, MAR)

A Quanta 200 FEG Environmental Scanning Electron Microscope (ESEM) provided by FEI Company (Eindhoven, The Netherlands) equipped with a field electron gun was used to perform the sample observations. Calibration of the microscope was performed with a standard grid (MRS3, Geller microanal. Lab, Topsfield, MA, USA). The Wet-STEM (Scanning Transmission Electron Microscope) is a specific stage that is attached to the ESEM. It allows the direct observation of nanoparticles that are dispersed in an aqueous solution [59]. The observation conditions were: acceleration voltage = 30 kV, working distance = 7 mm, temperature = 2 °C, and water vapor pressure =706 Pa. Particular precautions must be taken not to dry the sample during the pumping in the ESEM chamber. First, a 20 μL drop of the solution to be observed is deposited onto a holey carbon grid. Then, a pumping sequence is programed to replace progressively the air present in the chamber by the expected pressure of water vapor. When this limit was reached, the water vapor in the ESEM chamber was in equilibrium with the liquid sample where the temperature was 2 °C according to the water phase diagram. Thus, the sample must be thinned sufficiently to be transparent to the electron beam to observe the nanoparticles directly in the liquid layer. This was achieved by slightly decreasing the gas pressure in the chamber by a few tens of Pa. This yielded the controlled vaporization of water, and a thinning of the sample thickness. When the transparency to the electron beam was obtained, the gas pressure was kept at 706 Pa, the exact pressure necessary to maintain the equilibrium between the liquid and gaseous phases. Sample observation was preferentially performed through the holes of the holey grid. Indeed, within holes, nanoparticles were expected to remain within the liquid without any contact of the particles with the carbon layer. Mobility of nanoparticles also indicates a lack of contact with the carbon layer. One main limit of this technique was that the thinning of the sample was obtained by evaporating a part of the liquid phase. This yielded an increase of the nanoparticle concentration in the sample. To overcome this difficulty, the sample was first diluted with pure water (500–1000 times) before to be concentrated approximately to the initial concentration during the thinning of the liquid layer. When the sample was ready to be observed, i.e., when the nanoparticles were in the aqueous phase, images were continuously recorded with a scanning frequency ranging from 4 to 60 images per minute, depending on the motion of the nanoparticles. Several zones of the sample were observed successively to show the reproducibility of the observations. Size measurements were performed with Fiji [60].

### 2.6. STEM-in-SEM (STEM, MAR)

After being observed in the Wet-STEM mode, the sample was dried under vacuum to be observed with STEM-in-SEM mode. Dark field and bright field images of the nanoparticles initially dispersed in the liquid and then stuck on the carbon film of the holey carbon grid could be recorded with a 1 nm resolution. The acceleration voltage was 30 kV.

### 2.7. SEM (MAR)

Samples were deposited on a carbon stub and dried before introduction in the SEM chamber. Images were recorded using an acceleration voltage of 20 or 30 kV. Sample size measurements were performed using Fiji Software (version 1.49, NIH, Bethesda, MD, USA). The size measurements were calibrated by using both a calibration grid (Geller reference standard MRS-3XYZ with mount serial No. R30-147) for the SEM mode and a 204 nm latex spheres (202/197 nm and 205/201 nm) for the STEM mode.

### 2.8. SEM (SAC)

SEM images were obtained with a Carl Zeiss “Ultra 55”. The samples were deposited on a grid (same preparation as TEM (SAC) or directly on an adhesive membrane.

### 2.9. Small-Angle X-Ray Scattering (SAXS, MAR)

SAXS experiments were performed on a set-up operating in transmission geometry. A Mo anode associated to a Fox2D multi-shell mirror (XENOCS) delivered a collimated beam of wavelength 0.710 Å. Two sets of scatterless slits [61] delimited the beam to a square section of side length 0.8 mm. A MAR345 imaging plate detector allowed simultaneously recording scattering vectors q ranging from 0.25 nm^−1^ to 25 nm^−1^, meaning that distances up to 25 nm could be probed with this set-up. Samples were placed in glass capillaries of diameter 2 mm. Absolute intensities were obtained by measuring a calibration sample of high-density polyethylene (Goodfellow, Huntingdon, UK) for which the absolute scattering was already determined.

SAXS measurements on Ludox samples were carried out after a factor 100 dilution in water. This was done to ensure a dilute regime compatible with the observation of typical form factors of the nanoparticles, and to minimize the structure factor contribution arising from nanoparticle interactions. Calculations of monodisperse spherical form factors were made using the SASFit [62] software and were compared with experimental data. The diameter refinement was performed to obtain the best agreement with experimental data, in particular regarding the position of intensity minima and maxima. Reliability ranges corresponding to acceptable sphere diameters were estimated for each sample.

### 2.10. Dynamic Light Scattering (DLS, SAC)

For samples purchased as suspensions, DLS measurements were directly performed on a Horiba nanoparticle analyzer SZ 100. Before any measurement, the calibration is checked with the Nanosphere standard. If needed, concentration was further adapted to allow a proper measurement. For example, for the measurement of nanosphere standard, 2–3 drops of suspension were mixed with pure water. When samples were powders, they were dispersed in water at a temperature regulated at 18 °C for 20 min (2 s on, 2 s off) at 30% power, using an ultrasonic cuphorn. NP average sizes and standard deviations were provided by the analyzer software.

### 2.11. Analysis of Particle Sizes

For most techniques, the size of nanoparticles (NPs) represents their diameter, except for AFM, in which size usually represents the height. In this study, all NPs used, except TiO_2_ E171, are pseudo-spherical and thus the height and diameter values of the NPs are comparable. To simplify the text, we only use the term “size” to represent the equivalent diameter or height of NPs; values are given as means ± standard deviations (SD) in nm for a given technique, whereas global averages are given as means of each technique ± standard error of the mean (SEM) in nm. The amplitude percentage variation is the ratio between the difference of the largest and smallest values over the global mean. Normality tests (D’Agostino–Pearson omnibus and Shapiro–Wilk) were performed within GraphPad Prism 5.0 with an alpha threshold of 0.05.

## 3. Results

The main theme of this intercomparison was to perform measurements at local laboratory facilities using daily used in-house protocols. Thus, no specific measurement protocol was designed as usually found in classical metrology study [41] and detailed uncertainties were not requested [63]. The goal was to perform experiments as close as possible to day-to-day practice so that interpretation of results could help us to present practical guidelines for measuring nanoparticle sizes at local facilities. The experiment was divided in three steps: (1) use available techniques to perform measurement on a standard system (polystyrene beads) for which the size was certified; (2) run experiments will all the available techniques on silicate NPs (Ludox®) which are homogeneous solutions but of unknown size; and (3) run experiments on two toxicology-important nanoparticle families: silver and titanium dioxide.

### 3.1. Size Measurement of a Standard

**Figure 1:**
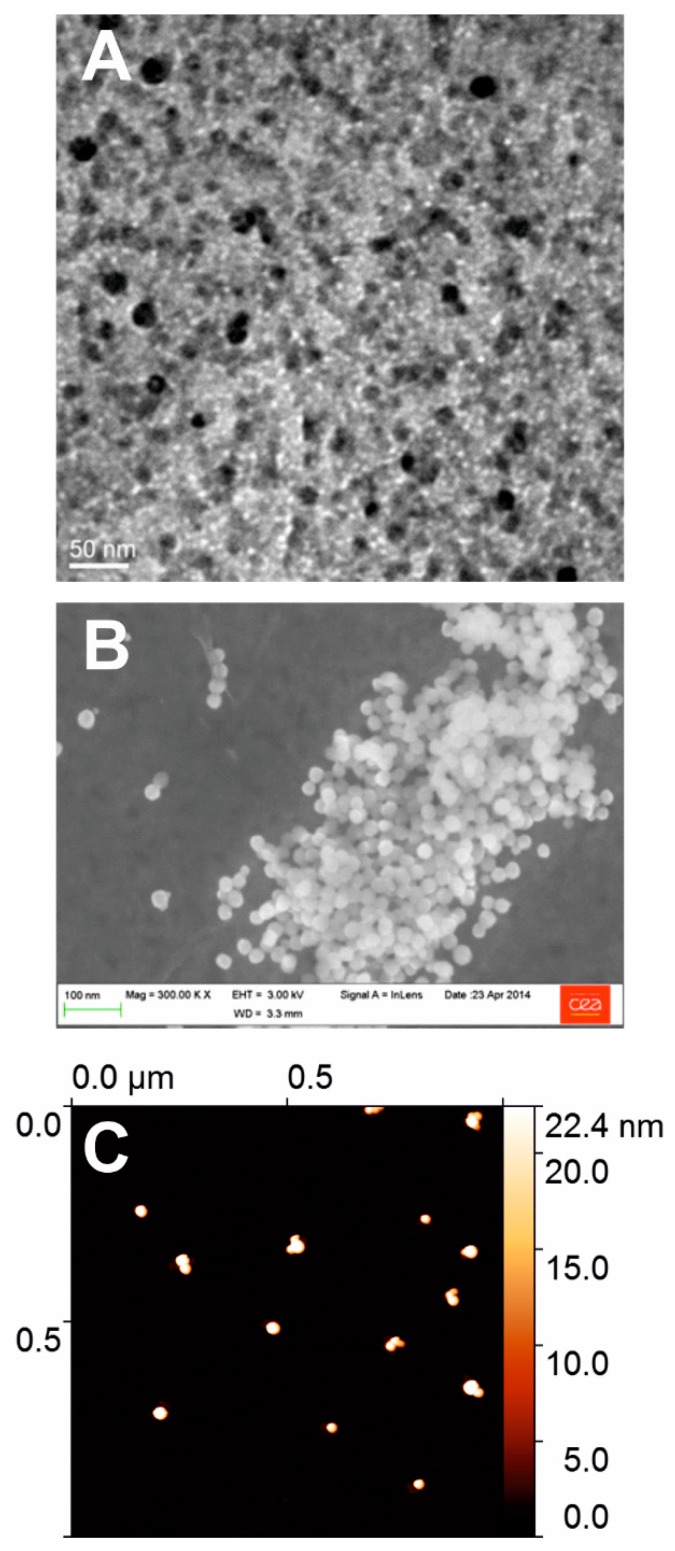
Gray scale images of nanosphere™ standard polystyrene particles of 22 ± 2 nm in diameter: (**A**) black particles in TEM image of 0.48 × 0.48 μm²; (**B**) white particles in SEM image of 1.0 × 0.67 μm²; and (**C**) white particles in AFM height image of 1 × 1 μm² where the height scale is represented by a false-color gradient on the right-hand side.

The nanosphere standard polystyrene particles (called PS22) were certified at 22 ± 2 nm diameter by the manufacturer (using DLS). Only four out of the six biophysical techniques were able to provide measurements. According to the limitation of local experimental set-ups, no data were obtained with SAXS and wet-STEM. SAXS could not provide a determination of the NP diameter due the too small electronic contrast between PS22 and water. The signal-to-noise ratio therefore remained too small even after several hours of measurements to reveal the mean particle size. Images collected by microscopy techniques (Figure 1) show densely packed beads of apparently homogeneous size.

**Figure 2:**
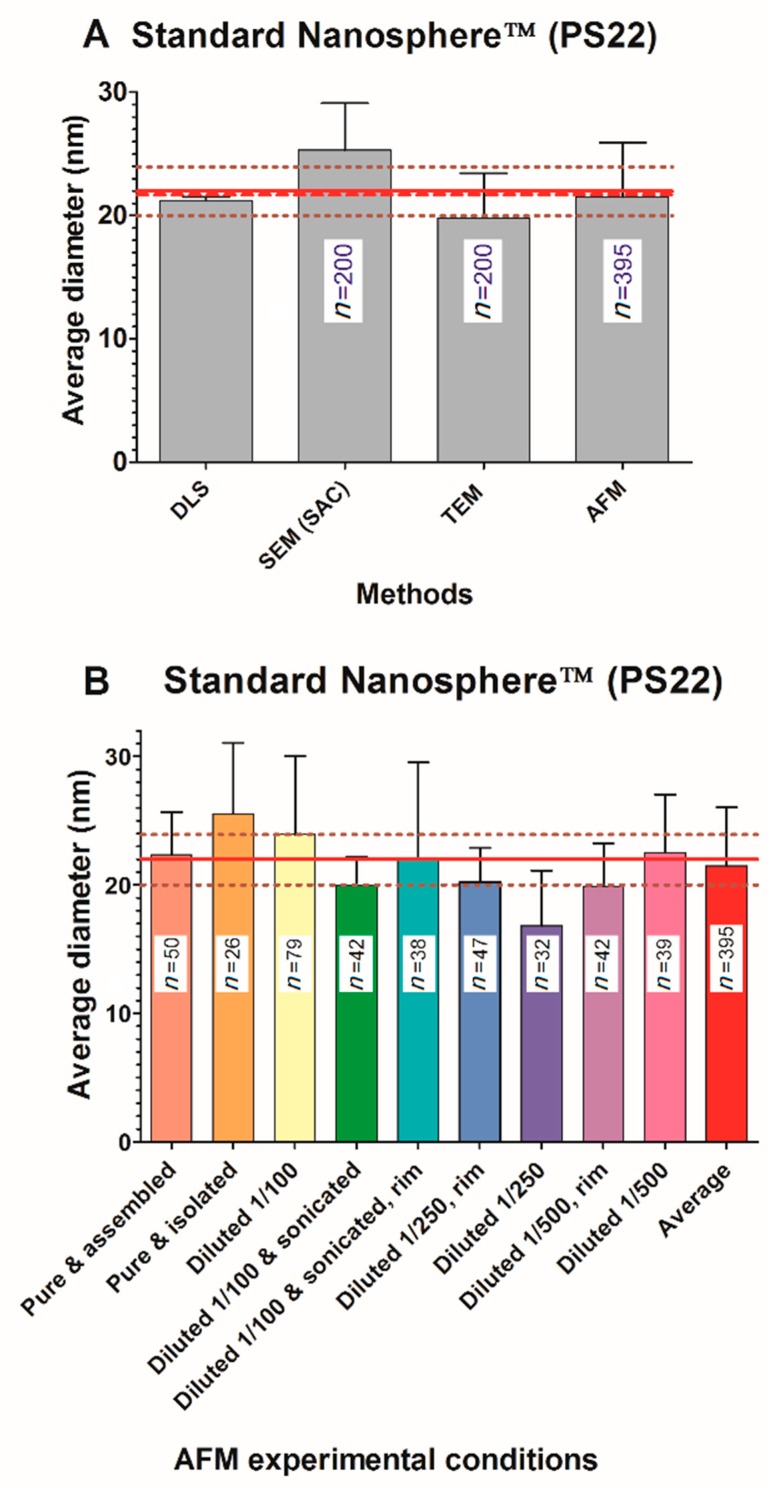
Size measurement of nanosphere standard particles (PS22) given at a reference diameter of 22 nm (**thick red lines**) and a standard deviation of ±2 nm (**dotted red lines**). N represents the number of measurements obtained in each method. (**A**) Measures were performed with Dynamic Light Scattering (DLS), Scanning Electron Microscopy (SEM) in Saclay (SAC), Transmission Electron Microscopy (TEM), and Atomic Force Microscopy (AFM). The red dashed line corresponds to the average value of all the five applied methods (for visibility, standard deviation is not shown). (**B**) Size measurements performed solely with AFM using nine different experimental conditions where Pure indicates no dilution, Diluted indicates dilution in pure water, assembled indicates that size was obtained by measuring the length of aligned PS22 and divided by the number of particles, sonicated indicates that sonication was performed on the sample before imaging, and rim indicates that measurements were performed at the edge of deposited particles on mica.

Average size measurements of PS22 are shown in Figure 2A. The global average value of four tested techniques is 22.0 ± 1.2 nm (red dashed line, Figure 2) where the SEM has the largest deviation. Let us note that 20 nm is a small size for SEM; the precision of the measurements is therefore questionable. Surprisingly, average size of PS22 (19.8 nm) determined by TEM is quite below the reference value, although PS22 size is within the TEM standard deviation. Concerning TEM as well as SEM, the contrast of PS22 NPs was rather poor and may explain the noted over/underestimation of the size. Interestingly, the average value of all the tested techniques is much closer to the reference value than most of individual techniques.

Size determination of PS22 with AFM appears to be the most accurate (average size = 21.5 ± 4.6 nm). It should be noticed that this average value is itself an average value of several different AFM experiments (Figure 1B). AFM data consisted of 395 measurements performed on nine different and independent experimental conditions using various size measurement approaches (see Materials and Methods and Figure S5). Heterogeneity in AFM measurements seemingly resembles those observed in average values from different techniques with minimum and maximum values ranging from 17 to 25 nm in both cases, although AFM data show a larger standard deviation. Globally, results obtained on PS22 are within a 25% variation (percentage amplitude) among the four tested biophysical methods. Remarkably, the extreme variations cancel out to provide a global average value equal to the expected calibrated value. It should be noted that none of the differences among the four methods are statistically significant according to a one-way analysis of variance (ANOVA) test (GraphPad Prism 5.0, USA).

### 3.2. Size Measurements of Monodisperse Silicate Solutions

**Figure 3:**
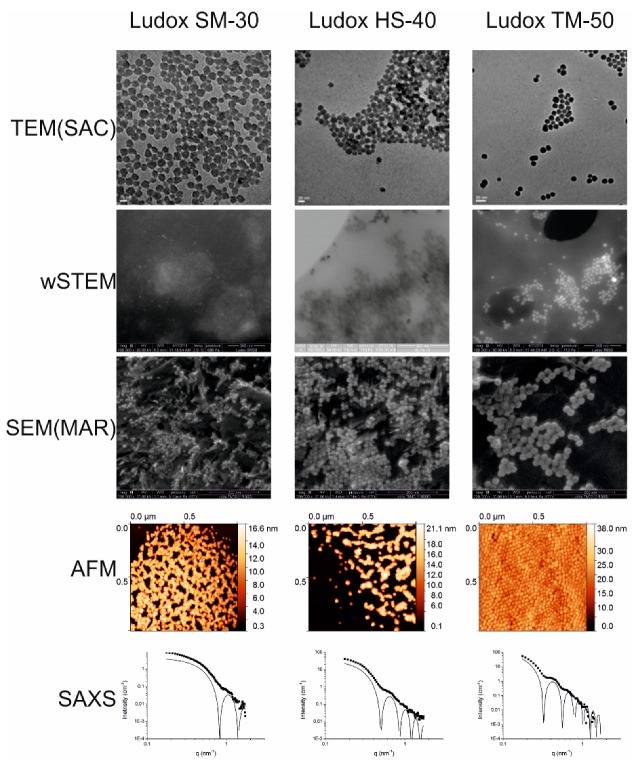
Typical experimental measurements of monodisperse silicate solutions (Ludox). According to the manufacturer’s documentation, the estimated size of SM-30 is 7 nm, HS-40 is 12 nm, and TM-50 is 22 nm. Experimental data include Transmission Electron Microscopy (TEM), wet Scanning Transmission Electron Microscopy (wSTEM), Scanning Electron Microscopy (SEM), Atomic Force Microscopy (AFM), and Small-Angle X-ray Scattering (SAXS). Scale bars are given on the EM images or on the x-axis and y-axis of AFM images. Height color scales are shown on the right of AFM images.

**Figure 4:**
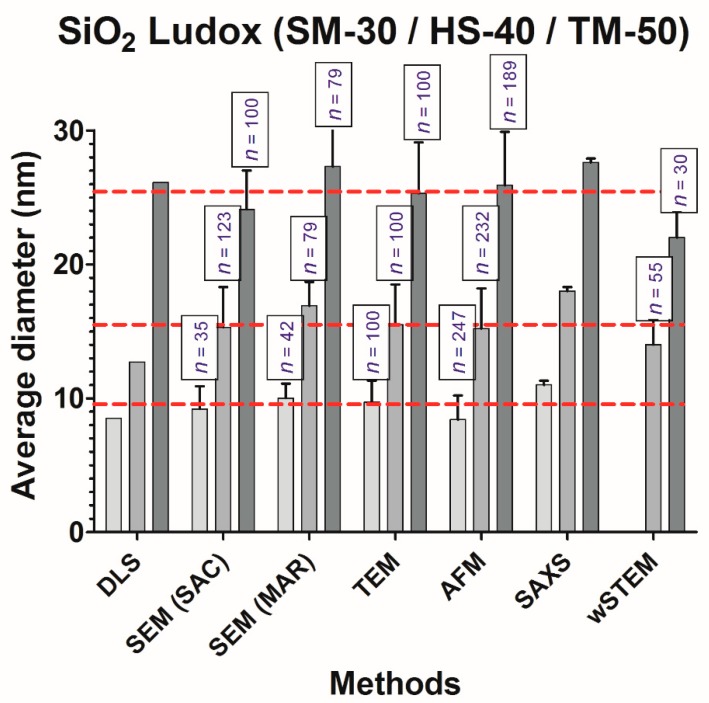
Size measurement of three monodisperse solution of SiO_2_ nanoparticles (Ludox). Measurements were performed with Dynamic Light Scattering (DLS), Scanning Electron Microscopy (SEM), Transmission Electron Microscopy (TEM), Atomic Force Microscopy (AFM), Small-Angle X-ray Scattering, and liquid Scanning Transmission Electron Microscopy (wet-STEM). Results on Ludox SM30/HS40/TM50 are shown in light/medium/dark gray colors, respectively. The red dashed lines correspond to the average values of all the applied methods: 9.3 ± 1.5 nm for SM30, 15.6 ± 2.6 nm for HS40, and 25.4 ± 2.9 nm for TM50. *n* represents the number of measurements obtained in each method.

Ludox colloidal silicate solutions were chosen due to their low agglomeration. This choice was made during the COST Action TD1002. Examples of images obtained by all tested techniques are shown in Figure 3 and averaged size values are represented in Figure 4. Unfortunately, Ludox manufacturer does not provide average sizes; consequently, we used our global average values (red dashed line in Figure 4) as an internal reference, i.e., 9.5 ± 0.4 nm (*n* = 6), 15.4 ± 0.7 nm (*n* = 7), and 25.5 ± 0.7 nm (*n* = 7) for Ludox SM30, HS40, and TM50, respectively. On the three Ludox solutions, TEM has the smallest difference with our internal reference definition (global averages), followed by AFM, SEM, DLS, and SAXS. The SAXS technique provides average sizes of silicate systematically higher than any other technique. This might be due to the dependence of the SAXS intensity with the volume of the scattering particles, which might result in a slight overestimation of the mean diameter of a collection of spherical objects. Ludox SM30 are slightly too small or too mobile to allow for an accurate size measurement using wet-STEM. Surprisingly, all SEM data from one laboratory have average values lower than our defined reference (red-dashed line), while all SEM data from the other laboratory have average values higher. Although several causes could be found (see below with the operational aspects), the differences between these values are within the standard deviations of the measures. The amplitude percentage variations between all the direct techniques (SEM, TEM, AFM, and wSTEM) for the Ludox SM-30, HS-40, and TM-50 are 17%, 19%, and 21%, respectively, whereas they are 27%, 34%, and 22% for all techniques, respectively. Although the amplitude percentage variation increases when indirect methods are added, there is no significant differences between all the mean values according to a one-way ANOVA test (GraphPad Prism 5.0). Similar to the reference PS22 material, results obtained on Ludox NPs indicate a global variation less than 24% among the six tested biophysical techniques, which validates each of those for measuring isolated nanometer-scale particle sizes.

### 3.3. Size Measurements of Silver Nanoparticles

**Figure 5:**
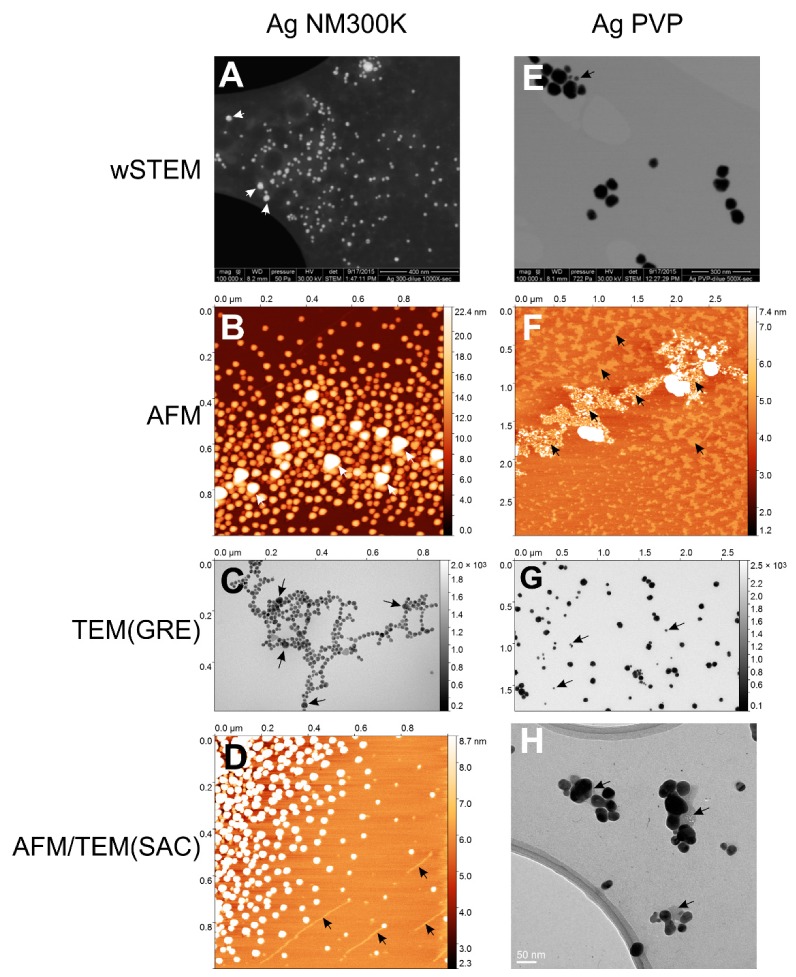
Samples of recorded microscopy images on silver NPs: (**A**–**D**) for Ag NM-300K; and (**E**–**H**) for Ag PVP. Whereas the global average of NM-300K size is 17.1 ± 3.5 nm, there is a presence of significantly larger NPs (indicated with black or white arrows) around 50 nm as observed in: wSTEM (dark field) (**A**); AFM (**B**); and TEM (**C**). Illustration of silver NPs stability as observed with AFM (**D**), where weakly bound NM-300K NPs were pushed around by the AFM tip (silver traces are indicated with black arrows) which results in a noticeable reduction in NPs height. Conversely, whereas the global average of PVP size is 55.8 ± 3.7 nm, there is a presence of smaller NPs (marked with black arrows) in wSTEM (**E**) and TEM (**G**) images. In AFM (**F**) and TEM(SAC) (**H**), the coating layer of PVP surrounding silver NPs is clearly seen (marked with black arrows), which is one explanation of the smallest average NPs size determined by AFM. Note that the color scale has been adjusted for the AFM data (**F**) so that the small height of PVP remains visible while NPs appear in bright white colors.

**Figure 6:**
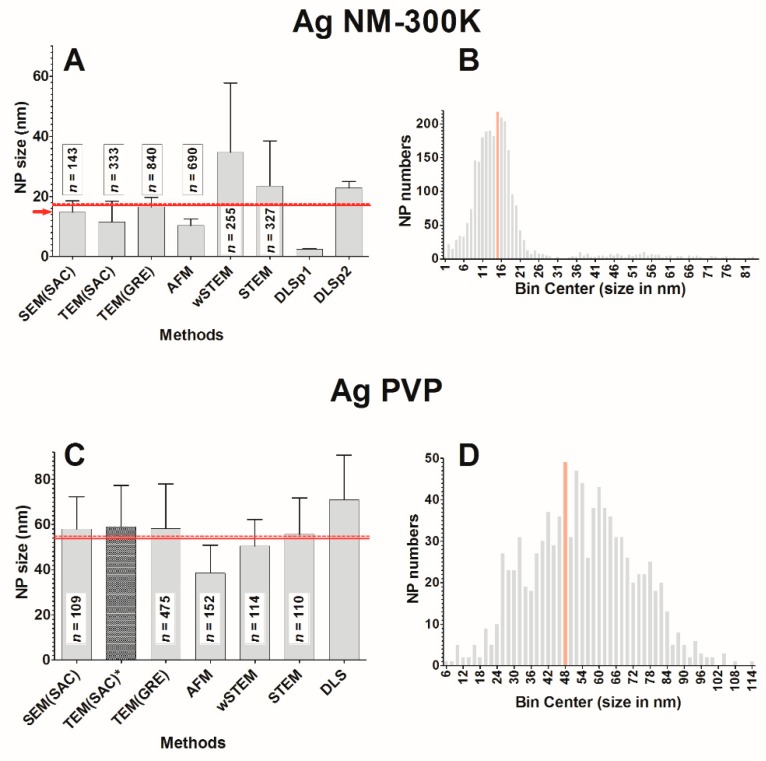
Average size measurements for Silver NPs: (**A**) Ag NM-300K; and (**C**) Ag PVP; and their respective frequency distance histogram distributions: (**B**) Ag NM-300K; and (**D**) Ag PVP. Methods are Dynamic Light Scattering (DLS), Scanning Electron Microscopy (SEM), Transmission Electron Microscopy (TEM), Atomic Force Microscopy (AFM), wet Scanning Transmission Electron Microscopy (wSTEM), and Scanning Transmission Electron Microscopy (STEM). The difference in the sample between wSTEM and STEM is the presence of liquid in the former, whereas it is dried in the latter. When two distinct populations of NPs are observed, they are reported with p1 and p2 keywords. The red lines in (**A**,**C**) indicate the global average NP size values determined by direct techniques only, 17.0 nm for NM-300K, and 53.9 nm for Ag PVP, whereas the dashed lines indicate the global NP size values determined by both direct and indirect techniques, 17.1 nm for NM-300K, and 55.8 nm for Ag PVP. The red arrow in (**A**) indicates the size of the NM-300K narrow population determined in the JRC report. The principal mode values are highlighted in pink on the frequency distance histograms: 15 nm for Ag NM-300K and 48 nm for Ag PVP. The * indicates that the value has been published earlier [47].

Two silver (Ag) nanoparticles were selected: NM-300K and PVP. Ag NM-300K is a European reference material where 99% of NM-300K particles have a size less than 20 nm, while 1% is above 20 nm [51]. Polyvinylpyrrolidone (PVP) is a surfactant that allows a greater dispersion of silver particles; the PVP-coated silver NPs do not have a reference size but it was measured previously at 59 ± 18 nm [47] using TEM. Images of both silver nanoparticles are shown in Figure 5 and size measurements are summarized in Figure 6. More than 99% of all silver NPs have a size <100 nm (Table S1).

The average size of NM-300K NPs for each technique is shown in Figure 6A. When pooling all the data from direct methods (SEM, TEM, AFM, wSTEM, and STEM), the average size of NM-300K is 17.0 ± 12.3 nm (red line, Figure 6A) with a median value at 14.5 nm. When averaging the mean of all techniques (direct and indirect) the global size is 17.1 ± 3.5 nm (*n* = 8, dashed line, Figure 6A). Despite the large number of measured NPs (***n*** = 2588), the two performed normality tests failed (GraphPad Prism 5.0). The amplitude percentage variation for direct techniques is 145%, whereas it is 188% with all techniques. These variations, as well as the high SD value of direct methods, strongly suggest heterogeneity in the NM-300K NPs. The frequency distance histogram of all the direct techniques reveals a multimodal size distribution for NM-300K (Figure 6B). The principal mode is at 15 nm, whereas minor modes are observed at 2, 37, and 54 nm. Indeed, DLS results indicate the presence of two populations (around 2.5 and 22.8 nm), which is confirmed by a careful TEM(SAC) study that identifies two populations around 4.2 and 14.9 nm. Results for the TEM(SAC) column concatenate both populations (Figure 6A). In addition, the surprisingly long tail in size distribution toward large NP sizes indicate the presence of large NM-300K NPs, as observed with some techniques such as wet-STEM and STEM (Figure 5A), which clearly noticed the presence of two main populations around 15 and 57 nm, and 15 and 53 nm, respectively. Liquid imaging of NM-300K NPs with wet-STEM did not reveal any agglomeration during image recording (Figure S1) and the drying stage seen in STEM did not change the presence of large NPs (Figure S2). Large NPs were observed with TEM and AFM techniques (Figure 5B,C). In the former one, the low abundance of large NPs did not impact the SD value, whereas in the latter, large NPs were observed on top of smaller ones causing the impossibility to determine accurately their size (by height) and therefore were not counted with AFM. Variability in NM-300K size can also be due to degradation as evidenced with AFM imaging which is the likely cause of the reduced average value observed in AFM (Figure 5D). This is a well-known problem as Ag° degrades over time since it is not at equilibrium in complex environment [64,65].

The average size of PVP-coated silver NPs for each technique is shown in Figure 6C. When pooling all the data from direct methods (SEM, TEM, AFM, wSTEM, and STEM), the average size of Ag PVP is 53.9 ± 18.3 nm (red line, Figure 6C) with a median value at 53.2 nm. When averaging the mean of all techniques (direct and indirect) the global size is 55.8 ± 3.7 nm (*n* = 7, dashed line, Figure 6C). The amplitude percentage variation for direct techniques is 38%, whereas it is 58% for all techniques, which would have been very close to values obtained for monodisperse NPs (PS22 or Ludox) if we excluded AFM data (16% and 37% for remaining direct methods and all remaining methods, respectively). Indeed, similar to NM-300K NPs, the average size of Ag PVP is much lower for AFM data (38.5 ± 12.4 nm) than for all other direct methods. The degradation of NPs is likely a cause while the disruption of the PVP coating is clearly observed in AFM and TEM images (Figure 5F,H). Images of a wet-STEM time series clearly show the motion of PVP-coated silver NP during imaging (Figure S3). It also shows that these NPs are not agglomerated in solution and, after drying, it is also possible to detect the presence of a smaller-size population (Figure 5E), similar to what is observed with TEM (Figure 5G). The possible degradation of PVP NPs with or without combination with the disruption of the PVP coating is likely responsible for the large SD values obtained by all the tested techniques. It is thus not surprising that both normality tests failed for all combined direct technique measurements (***n*** = 960). The frequency distance histogram of all the direct techniques reveals again a broad size distribution (Figure 6D), which was not anticipated from individual results since every technique reported a monomodal distribution (Table S1). The major mode appears at 48 nm while minor modes can be observed at 32, 58, 78, and 88 nm.

### 3.4. Size Measurements of Titane Dioxide Nanoparticles

**Figure 7:**
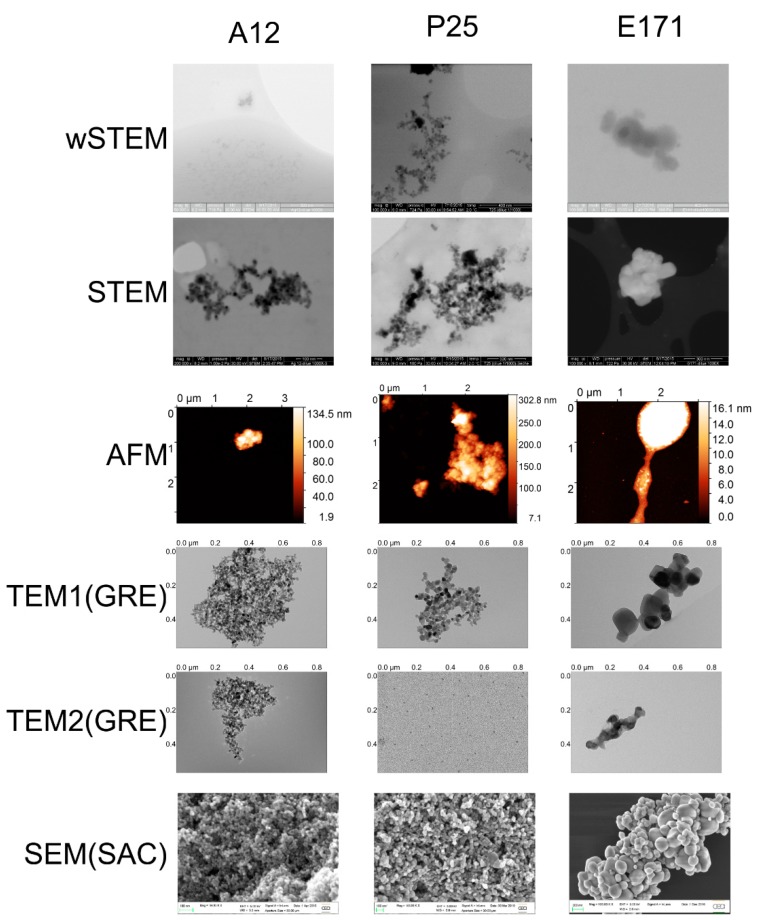
Samples of recorded microscopy images on TiO_2_ NPs. Images show that, all three NPs, A12, P25, and E171, appear agglomerated in all techniques. Scale bars are indicated on the wSTEM, STEM or SEM images, whereas they appear on top of AFM or TEM images. The AFM image for E171 has been saturated to better visualize smaller NPs present in this sample. The difference between TEM1 and TEM2 images is in the method used for depositing samples: carbon floatation technique for the former or directly onto a carbon-coated grid for the latter (see methods).

Three TiO_2_ NPs were selected for their interest in nanotoxicology. Suspensions of TiO_2_ (P25) were obtained after strong sonication of commercial products; primary particle size of P25 is given around 21 nm from the manufacturer and a recent analysis reports a value of 23 ± 10 nm [49]. Due to the high temperature synthesis of P25 NPs, they are constituted of two kinds of particles: 75% of anatase, and 25% of rutile. An initial report using TEM indicates that the average size of anatase is 85 nm, whereas that of rutile is 25 nm [66]. TiO_2_ A12 was determined around 12 nm in size [67]. Food additive E171 was directly purchased from a manufacturer as a white powder; size of E171 is unknown but usually advertised as above 100 nm in diameter; it is therefore not considered as a nanomaterial in consumer products. A major difference with previously presented NPs is the strong tendency of all TiO_2_ NPs to agglomerate, in both liquid and dry conditions (Figure 7), especially for P25 and A12 NPs, which are synthesized by gas phase methods (flame spray or laser pyrolysis) usually producing agglomerated materials.

Since the three TiO_2_ NPs were provided as powders, specific surface areas were determined using the BET method: 82 m²/g for A12 (with the density of 3.9; the estimated diameter is 19 nm), 46 m²/g for P25 (with the density of 3.9; the estimated diameter is 33 nm), and 9.4 m²/g for E171 (with the density of 4.0; the estimated diameter is 160 nm). Variations between BET results and global average values from this study range from 21% to 43% (Table S1).

**Figure 8:**
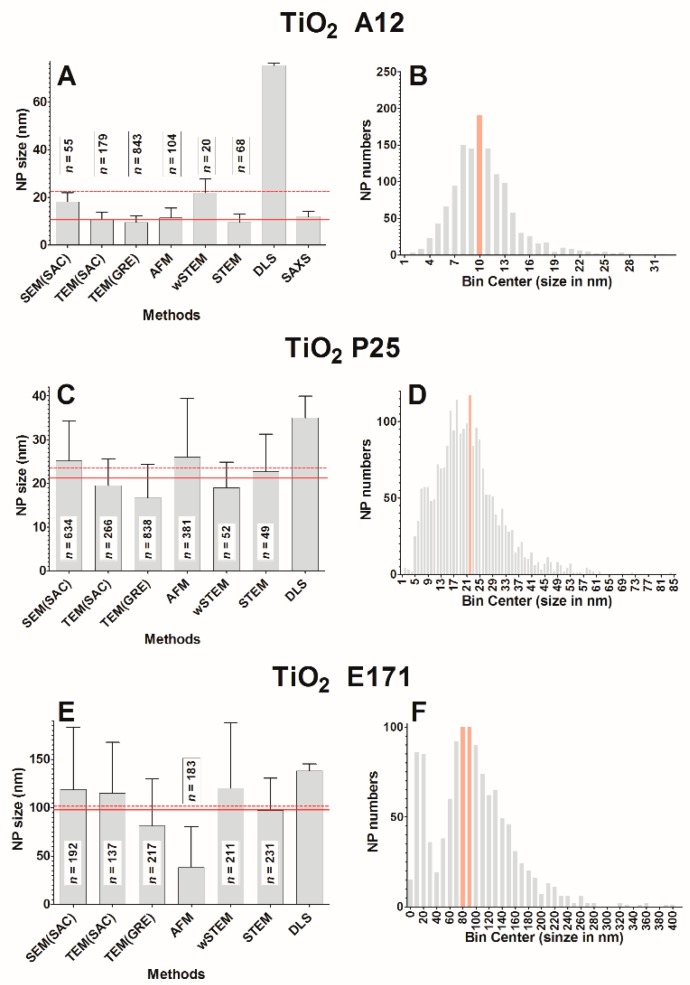
Average size measurements for TiO_2_ NPs: (**A**) A12; (**C**) P25; and (**E**) E171; and their respective frequency distance histogram distributions: (**B**) A12; (**D**) P25; and (**F**) E171. Methods are the same as in Figure 6 plus Small-Angle Xray-Scattering (SAXS). The red lines in (**A**,**C**,**E**) indicate the global average NP size values determined by direct techniques only, 10.5 nm for A12, 21.3 nm for P25, and 94.8 nm for E171, whereas the dashed lines indicate the global NP size values determined by both direct and indirect techniques, 22.5 nm for A12, 23.5 nm for P25, and 101.3 nm for E171. The principal mode values are highlighted in pink on the frequency distance histograms: 10 nm for A12, 22 nm for P25, and 85 for E171.

The average size of TiO_2_ A12 for each technique is shown in Figure 8A. When pooling all the data from direct methods (SEM, TEM, AFM, wSTEM, and STEM), the average size of A12 is 10.5 ± 3.8 nm (red line, Figure 8A) with a median value at 9.9 nm. When averaging the mean of all techniques (direct and indirect), the global size is 22.5 ± 8.5 nm (*n* = 8, dashed line, Figure 8A). A total of 100% of A12 NPs have a size below 100 nm. Due to the agglomeration of A12 NPs, the DLS-estimated size is very different from other techniques including the indirect SAXS method. It should be remembered that the SAXS instrument used in this study has a cutoff around 30 nm and thus any particles larger than this threshold are not seen. The value of 12 ± 2.1 nm from the SAXS technique implies that there must be a significant proportion of isolated NPs in the A12 solution. The frequency distance histogram of all the direct techniques reveals a monomodal size distribution for A12 NPs (Figure 8B); however, despite the large number of measurements (*n* = 1269), both normality tests failed. The major mode is at 10 nm. The amplitude percentage variation for direct techniques is 119%, whereas it is 293% for all techniques. Such variations appear in contradiction with the monomodal distribution of determined NP sizes. If we exclude the value of DLS, the large amplitude variation highlights the precision of the size measurement itself. Because of the agglomerated state of A12 NPs, it is complicated for AFM images to measure height values (only NPs at the border of the agglomerate can be analyzed) and it is similarly challenging to delimit the boundaries of agglomerated NPs using EM methods. In addition, as mentioned earlier, the size of 10 nm is at the technical limit for SEM-based methods. Another indication of the difficulty of measurements comes from the comparison of the pooled average size of 1269 A12 NPs (10.5 nm) with that of the average of each direct technique mean values (13.6 nm, see Table 1). Except for TEM(GRE), most techniques provided a low number of measurements (near or less than 100) which could also explain the large amplitude variation.

**Figure 9:**
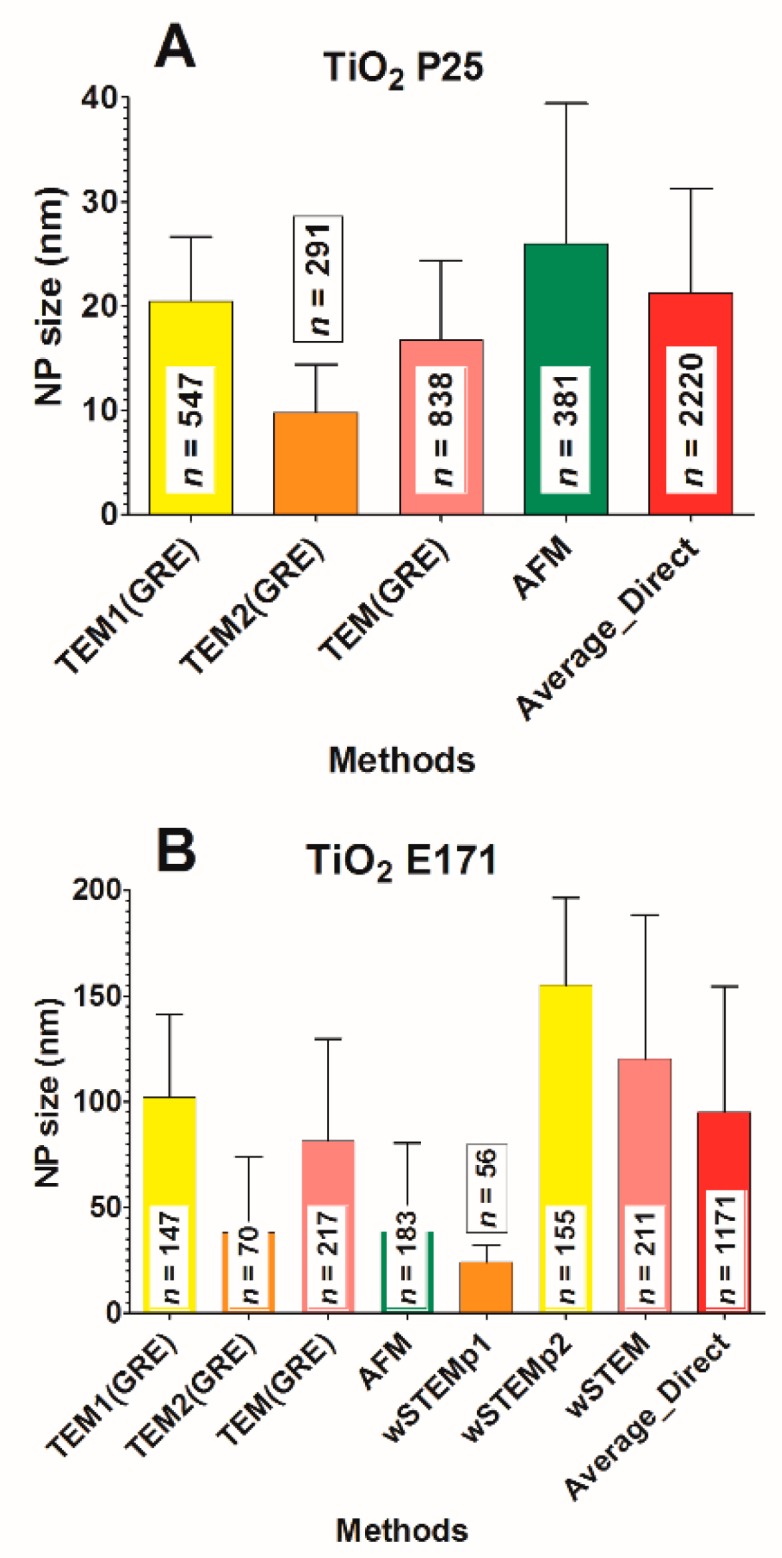
Average size measurements for sub-populations of TiO_2_ NPs: (**A**) P25; and (**B**) E171. The larger size populations are colored in yellow, whereas smaller size populations are in orange. Global average of NP sizes within the same technique is colored in pink. The large SD values in AFM data (green columns) prompted checking for the presence of smaller NPs in TEM data by changing the protocol of NPs deposition (TEM1, carbon floatation; and TEM2, direct deposition; see Materials and Methods). For both NPs, the TEM2 method identified the presence of smaller NPs than with the TEM1 method, in close agreement with AFM results (which is using a direct deposition method). The global average value of all tested direct techniques is shown in the last column (in red). The two sub-populations observed in wet-STEM were described prior to the change of TEM(GRE) protocol.

The average size of TiO_2_ P25 for each technique is shown in Figure 8C. When pooling all the data from direct methods (SEM, TEM, AFM, wSTEM, and STEM), the average size of P25 is 21.3 ± 10.0 nm (red line, Figure 8C) with a median value at 20.2 nm. When averaging the mean of all techniques (direct and indirect), the global size is 23.5 ± 2.3 nm (*n* = 7, dashed line, Figure 8C). A total of 100% of P25 NPS have a size below 100 nm. Similar to results obtained with the PVP-coated silver NPs, the homogeneity in global size averages and the large SD values suggest the presence of heterogeneous P25 NPs. The frequency distance histogram of all the direct techniques reveals a multimodal size distribution (Figure 8D) with a major mode at 22 nm while minor modes can be observed at 8 or 32 nm. Similar to the TiO_2_ A12 and likely due to agglomeration, DLS average value (35 ± 5 nm, [68]) is above every other techniques. The amplitude percentage variation for direct techniques is 40%, whereas it is 77% for all techniques. The broad distribution of P25 sizes near smaller sizes (with a minor mode 12 nm) raised the question why such small size NPs could not be observed in initial TEM measurements (called TEM1). It was decided to perform a second TEM analysis (called TEM2) where the sample was deposited directly on carbon-coated grids, similar to what is performed in AFM. Surprisingly, in TEM2 data, mostly small and isolated P25 NPs (9.8 ± 4.6 nm) were observed on the carbon grid (Figure 9A). Such results reveal a true operational aspect in NP size determination where small NPs were primarily retained by the mica surface in TEM1 experiment (see methods) and thus absent from the floating carbon layer. The apparent higher binding of small TiO_2_ NPs on mica (negatively charged surface) is consistent with a lower isoelectric point value for smaller TiO_2_ NPs than with larger ones [69].

The average size of TiO_2_ E171 for each technique is shown in Figure 8E. When pooling all the data from direct methods (SEM, TEM, AFM, wSTEM, and STEM), the average size of E171 is 94.8 ± 59.5 nm (red line, Figure 8E) with a median value at 89.7 nm. From direct methods, 57 % of E171 NPs have a size below 100 nm (Table S1). When averaging the mean of all techniques (direct and indirect) the global size is 101.3 ± 12.5 nm (*n* = 7, dashed line, Figure 8E). Similar to all previous TiO_2_ NPs, adding the DLS value increases the global E171 size average. This is especially important since averaged size values are near the cutoff value used for a nanomaterial definition. TiO_2_ E171 is systematically observed as agglomerates and results for E171 also provide the largest SD values observed in our entire study, in agreement with a previous TEM characterization [50], although the global average size is lower in our study. The amplitude percentage variation for direct techniques is 86%, whereas it is 98% for all techniques. This large variation in amplitude is mostly due to AFM data that provide an average E171 size at 38.4 ± 41.8 nm. The frequency distance histogram of all the direct techniques reveals a multimodal size distribution (Figure 8F) with a major mode at 85 nm and a minor mode around 10 nm. Similar to P25 NPs, the observed low sizes with AFM prompted us to check another protocol for TEM imaging. Again, there is a strong operational aspect since the average E171 size determined with TEM1 method is 102 ± 39.2 nm, whereas it is 38.2 ± 35.8 nm with TEM2 method (Figure 9B); representative TEM images are shown in Figure 7. The wet-STEM technique also identified a small size E171 population around 24.2 ± 8.2 nm and a larger size around 155.1 ± 41.0 nm (Figure 9B).

## 4. Discussion

The main learning experience from such intercomparison is to present a statistically complete set of data for determining NP sizes accurately with in-house biophysical techniques. Detailed difficulties of each technique as well as suggested good practice can be found in the Supplementary Materials.

Let us briefly summarize the main characteristics of major techniques used in this study by classifying them into two categories: direct and indirect techniques.

Direct methods produce images onto which size measurements can be directly performed and where the uncertainties are determined from the size distribution. Electron Microscopies (EM) provide 2D images with a greater contrast for NPs with high atomic number elements and a resolution below 1 nm. Size measurements are usually performed manually (shape recognition software can also be used if the contrast is satisfactory) by identifying and labeling NPs on an image and by estimating the pixel length of their diameter. It is difficult to characterize aggregates with EM (lack of 2D contrast) and it is not sensitive to the organic capping often present on commercial NPs. Due to its higher resolution, TEM is often preferred to SEM for characterization of NPs with a size below about 20 nm. Wet-Scanning Transmission Electron Microscopy (wet-STEM) consists in implementing a dedicated wet-STEM stage in any Environmental SEM (ESEM). This approach allows the observation of NPs in liquid state at first, and when the liquid layer evaporates, dry NPs can be visualized. Although the thinning of the liquid film can be tricky during introduction of the sample in the ESEM chamber, it takes no more than 30 min from the grid preparation to sample observation (see Methods for more details). This technique allows the recording of images with a 3 nm lateral resolution at 30 kV [70], which makes possible the observation for most of the nanoparticles having a diameter higher than 6 nm. AFM, the other microscopy-based technique, produces images of a sample deposited on a flat substrate (usually mica or graphite). Images are obtained by rastering a nanosize tip over the sample which makes AFM a “touching” microscopy technique. AFM images encode at each pixel the height value of the probed sample area. Accordingly, AFM is the only direct technique that can provide a real direct three-dimensional size analysis of a single NP. AFM height values are characterized with a resolution below 1 nm; however, due to the tip convolution effect which distorts AFM images, AFM lateral sizes depends on the size and shape of the AFM tip, which always produced an overestimation of x-axis and y-axis dimensions. Depending on the applied force on the AFM tip, AFM technique can detect organic capping of NPs. Size measurements can be performed automatically if NPs are sufficiently isolated by taking the tallest height value of a NP of interest from the image; however, in the case of NP agglomeration, manual cross-section profile can be used to extract size values.

Indirect techniques require a physical model to extract NP size from the raw experimental signal. For light scattering techniques (e.g., DLS), the Raleigh scattering dictates that the light intensity scattered by a NP is proportional to its diameter raised to the sixth power; thus, large diameter NPs will dominate the intensity signal and even a low population of large particles can make difficult the detection and analysis of a population of small size. A polydispersity index >0.1 limit has been suggested, above which DLS can no longer be interpreted [39]. DLS uncertainty is based on measurement repeatability. Modern instruments provide the analysis directly within the controlling software by giving an average hydrodynamic radius for each detected population. SAXS technique is well developed for determining NP sizes of simple geometric shape, but the requirement for measuring at very low angle, for large particles, usually requires specific instruments not often found in common departments. Moreover, the objects should present a sufficiently high electronic contrast with the surrounding continuous phase. Measurement of SAXS uncertainty is often fixed at 10%, a value based on previous experiences in the NIST laboratory.

The goal of NP size characterization is to provide a statistical estimate of the average size of their primary elements. This is, however, not a trivial quest. Intercomparison experiments assume that all the tested techniques determine a similar measurand. According to the metrology definition, a measurand is the parameter to be measured. In this work, the goal was to measure the diameter of NPs (or their radius). However, all tested techniques in this work did not determine the same measurand: for direct methods (microscopies), it is a geometrical diameter (or radius), whereas, for indirect methods (light scattering or SAXS), it is rather a hydrodynamic radius. For mono-dispersed and spherical isolated NPs in solution, both geometric and hydrodynamic radii should provide very similar values. This is in agreement with our results when considering the non-agglomerated and monodispersed samples (nanosphere and Ludox series) where no significant deviations are observed between direct and indirect techniques (one-way ANOVA test for all NPs measured in this work).

The classical representation of a sample from a population is to provide a mean value and a standard deviation (SD) around the mean. Without considering the shape of the true underlying distribution, the mean and SD are sufficient in most cases where NPs are mainly spherical, isolated, and monodispersed. In this work, this was the case only for model NPs (PS22 beads or solubilized silicate NPs Ludox) where both direct and indirect techniques provide a good estimate of the average NP size. Unfortunately, results in this work were different from the “realistic” NPs (silver and titanium oxide), where a strong heterogeneity in size or in agglomeration was observed (see frequency distance histograms in Figure 6 and Figure 8). It should be emphasized that size heterogeneity may be native (present during the fabrication of NPs) or due to degradations, as observed for silver NPs (Figure 5). Alternatively, results could also be expressed as a range which has often been the case in recent reports for P25 NPs (30–40 nm [71] or 500–2400 nm (aggregates) [72]) or for E171 NPs (31–200 nm [73], 30–600 nm [74], of 50–250 nm [75]). Sometimes, a range is even not provided but instead the ratio of true NPs (<100 nm) [76]. Nevertheless, range values are difficult to handle when attempting to compare distribution in sizes. The other frequently used size measurement is the median. It has a superiority over the classical mean in that it is less sensitive to extreme values when the number of measurements is limited. Most computed medians on NPs match closely the values of their corresponding mean, except for E171. Indeed, TiO_2_ E171 is a special case since, with all techniques, the average size is 101.3 nm, whereas it is only 94.8 nm with direct techniques. In addition, it was found that 53% of E171 NPs have a size below 100 nm. Thus, according to our results, this particular lot of E171 is at the threshold of the NP definition and a deeper investigation would be required to make a final judgment concerning the classification of this lot of E171 as NPs.

Another challenge in providing a proper size description of NPs is to evaluate the need to describe sub-populations. All difficulties could be summarized with a single term: proportion estimation. In other words: Is it possible to quantify the proportion of mixed NPs simply by looking at a couple of images for direct techniques? This difficulty for direct techniques is alleviated for indirect ones since it is often possible to fit data (for instance, DLS and SAXS) using different models that may include various sub-populations. However, adding contributions to fit experimental data should be considered carefully: the increase of fitting parameters always results in a better agreement between calculated and experimental profiles, but can also result in misleading interpretations.

For a regulatory purpose, size characterization of NPs simply needs to answer the question whether at least 50% in number of primary particles have at least one external dimension size below 100 nm. Unfortunately, there is no existing method to answer such a complex question [77]. For instance, indirect methods are incapable of distinguishing primary particles from agglomerated NPs and direct methods cannot guarantee a sufficiently large sampling of NPs to ascertain the 50% threshold value. This is particularly critical for heterogeneous NPs (such as TiO_2_ E171) where the average size of primary particles is around the 100 nm threshold in diameter. For a toxicological purpose, size characterization of NPs needs to describe all the possible sub-populations of NPs to make sure that identified functional results are really correlated with true NPs. Indeed, as described in the Introduction, sometimes a couple of tens of nm in a NP size could make a difference in toxicological tests. Whatever the purpose, it seems that a frequency distance histogram is the best answer when NPs are heterogeneous (either in intrinsic primary particle sizes or in agglomerated shape), which were all the “realistic” NPs tested in this work (silver and TiO_2_). It is likely not the easiest way but the combination of several direct methods coupled with different experimental setups are most likely to avoid any mis-characterization of NPs sizes. From a histogram, it is possible to extract an average value, a modal value, and the amplitude of values. From our results, Gaussian curve fitting did not provide a better characterization since no frequency distance distribution followed normality. Sub-populations were mostly visually identified by locating gaps in the histograms; there is likely room for improvement. For strictly monodisperse NPs, indirect methods are the best choice due to their simplicity and a single confirmation with a single direct method will ascertain that the average value between indirect and direct methods match.

Operational aspects are unexpected consequences of particular choices of experimental setups and they have been observed numerous times [78,79,80] since their first description [81]. The best-known operational aspect in NP size determination is the physical state of NPs. Due to their chemical natures or due to the environment (presence of salts and proteins from buffers or culture media), NPs have been known to agglomerate and aggregate [8,13,16,26,29,35,39,43,45,65]. Besides, due to its high energetic adhesive forces, which contribute to adsorbing proteins or small molecules, the surface of a NP is rarely “bare” [4], especially in biological media. Therefore, their size increases/decreases upon the agglomeration/disruption status of a given NP. Several studies focus on sample preparation and sample homogenization, where many efforts are targeted to the dispersion problem of NPs [29,33], including in nanotoxicology studies [68], and encouraging results have been found for specific NPs [43,44,45,82]. Although methods exist to model multimodal distribution of NPs using light scattering and SAXS techniques [83], a recent study reveals that the observation of multimodal dispersion of isobutylcyanoacrylate-based NPs only succeeded by AFM, nanoparticle tracking analysis, and tunable resistive pulse sensing techniques [33], whereas no light scattering batch methods could reproduce the particle size distribution.

A second level of the impact of operational aspects lies in the experimental protocol. It has been shown that two of the major biophysical techniques used for NP size determination (TEM and DLS) were the most prone to artifacts [36]. Such a claim is not used to disqualify these techniques regarding NP size determination, but rather to encourage researchers to pay closer attention to the protocol used. Drying samples is a classical criticism of microscopy techniques (EM and AFM), although a recent analysis reports that dry conditions in AFM is not the major hurdle in determining the correct height of a virus [80]. In fact, the nature of the substrate used to deposit a virus has been found to be the most sensitive parameter toward proper height measurement with AFM [80]. Such phenomenon has again been proven in this work, where NPs sizes obtained from TEM results were very different according to the method used to deposit the sample. In the first case (TEM1), a competition between mica and carbon layer was possible, whereas, in the second case (TEM2), only the contact with a carbon layer was allowed. When the TiO_2_ NP does not have a significant adsorption preference between mica and carbon layer, such as the TiO_2_ A12 NPs, determined sizes remain very similar in both methods (Table S1). However, for TiO_2_ P25 and E171, a difference between this adsorption led to characterize a totally different spectrum of NP sizes (Figure 9). Interestingly, the wet-STEM technique reveals the presence of both small and large E171 NPs which implies that both sizes are simultaneously present in the sample (Figure 9). The distinction between wSTEMp1 and wSTEMp2 was made at the very beginning of the analysis without knowing the unexpected behavior of E171 with carbon layers and mica. A similar observation was observed for TEM(SAC) on NM-300K NPs, where depending on the EM grids, a majority of small Ag NM-300K NPs could be observed when the Lacey carbon grids (with holes) were used (Figure S4).

The third level of the impact of operational aspect depends on the perspicacity of the human operator as defined by Eisenhart. It mostly pertains to the identification of multimodal NP size distribution. The case of the Ag NM-300K NPs is the most interesting. This NM-300K NP is part of representative manufactured nanomaterials from the European Commission Joint Research Center (JRC). A report provides a description of this nanomaterial as “silver particles of about 15 nm size with a narrow size distribution of 99 % of the particle number concentration exhibiting a diameter of below 20 nm” [51]. The report mentions the presence of smaller NPs (~5 nm) only observed with TEM, whereas larger NPs (~50–60 nm) have been observed with both NTA and DLS techniques. In this work, both minor species of NPs were observed: DLS and TEM for the small sizes, and wSTEM, AFM, and TEM for large sizes. It should be added that the small population found in TEM(SAC) has been pursued after the JRC report mentioned the presence of such small NPs. As seen in Figure 5A, wSTEM (and also STEM) data clearly reveal the presence of a majority of large size NM-300K NPs and consequently it was initially reported as a specific sub-population of this NP. Large NM-300K NPs were also observed with AFM and TEM(GRE) (see Figure 5B,C), however, their proportion relative to the “normal” size NM-300K is small (less than 1% of NM-300K was larger than 25 nm according to TEM data). Consequently, by pooling all the TEM(GRE) measures together, the impact of large NPs became minor on the average NP size. In AFM, such large NM-300K NPs were not taken into account since it was not possible to accurately determine their height because these large NPs sat on top of other NPs. Compared to our NM-300K direct technique-based average size of 16.8 ± 12.0 nm (Table 1), a recent study using traditional and tabletop TEMs provides similar values albeit with a narrower range in the NP size determination of NM-300K, 17.2 ± 5.5 nm and 19.1 ± 3.3 nm, respectively [84]. Quantification of sub-population proportions is a major challenge for metrological characterization of nanomaterials. The main difficulty is first to sample a very large number of NPs and second to make sure that the protocol used for size determination does not introduce a systematic bias as found above in EM or AFM. There is possibly another “human operator” impact in NP size determination and it is best illustrated with the TiO_2_ A12 NPs. All tested direct techniques (Figure 8A) but two, TEM(SAC) and AFM, observed a normality in the distribution of NP size values. This property is not correlated with the population size in each technique (normality was observed in the range of 20–843 NPs). Surprisingly, when pooling all the direct techniques data, normality tests failed on a total of 1269 NPs. It is possible that a miscalibration of some instruments is the cause of such phenomenon, albeit unlikely since there is no consistent bias toward low or high values among all the tested direct techniques. Another possibility is a sense of “dejà-vu” when an operator unconsciously manually selects mostly particles that resemble one another. If all NPs of an image cannot be selected (due to some agglomeration or border locations), the selection of NPs should not be restrained to those having “good similarities” to others.

Finally, the fourth level of the impact of the operational aspects is time. As seen with silver NPs, degradation of NPs is interactively measured (Figure 5D), implying that their stability upon time is a major concern. Beside the time, there could be an instrument bias causing the degradation of silver NPs. Indeed, AFM technique uses a red laser to detect changes in the contact between the sample surface and the AFM tip as well as a white light for the camera which may increase the rate of degradation of silver NPs. Thus, for light-sensitive NPs such as silver, AFM technique should minimize lighting or use a “dark mode” with piezoelectric tuning fork imaging [85].

What are the recommendations for proper NP size determination? First, choosing different biophysical techniques should reduce artifacts present in each known technique (direct or indirect). The presence of at least one direct technique is strongly recommended. Besides, providing an average across several techniques is likely more robust than any single technique. When a single technique provides an unusually different size estimation (e.g., DLS on A12 NPs in this work), it is then important to look carefully in every technique to highlight potential operational biases and, if not, the outlier result should be discarded in the final average. It is equally important to know the technical limitations of each method and it is always safer to contact specialist operators. Second, it is critical to make enough measurements regarding direct microscopy techniques. For monodispersed NPs, a range between 100 and 300 is mostly adequate. Measuring more than 300 sizes did not change the shape of a monomodal distribution. However, for heterogeneous NPs, our study cannot provide an upper limit since results from apparent monomodal distributions could not reach statistical normality with up to 2000 measurements. Although the normality distribution is not a goal in NPs size determination, we suggest that a minimum of 300 values per identified mode be the target value in number of measures. Third, do not make an educated guess about the expected size of NPs as we have seen in our work that an inter-technique amplitude variation of 300% is possible. There are multiple reasons for a similar NP to display large variations in its size: choice of techniques, impact of operational aspects, operator variability, degradation, etc. Fourth, it is equally critical to modify the experimental protocols to detect minor sub-populations. Modifications include change of concentration, change of deposition time, change of deposition method, change of substrate for imaging techniques, change in measurement methods, change in measurement software, and change in operators. This is important because changing experimental conditions will limit the influence of operational aspects. For example, changing the imaging mode or the image substrate in AFM (or grids in EM) should clearly be counted as two different biophysical characterizations. Fifth, it is important to harmonize the number of NPs counted per physical record (in microscopy, one record is one image). For instance, if a large NP (agglomerate) breaks into smaller pieces, it could be counted as hundreds of small particles. When adding several hundred “artifacts” values into a statistic, it will necessarily bias the final size evaluation; instead, if an average image contains 50 particles, then it is a safe practice to only measure around 50 particles per image. It is also strongly recommended to include minority sizes (small or large in size) to detect minor sub-populations of a sample that may present a strong bias from image to image. Sixth, keep appropriate records of measurement periods as NPs are subject to deterioration upon time (as it was found for the silver NPs in AFM). If NP size determination is performed at the reception of NPs, it would be necessary to repeat the size measurements if biological experiments using these NPs are not concomitant with their reception.

## 5. Conclusions

Nanotoxicology research aims at determining the potential harm of a given nanoparticle, especially regarding their size. Attributing a toxicological effect to the proper range of NP sizes is a major challenge and it is likely that some controversies in the effect of NPs sizes on cells or tissues could be due to poor characterization of the NP solutions used in toxicological tests. It is therefore of major importance to provide careful statistical evaluation of the performance of biophysical techniques used to determine NPs sizes. In this study, we aimed at using “standard” laboratory instruments to the best of their capacity without any preparation constraints and therefore following local laboratory protocols. Final NPs size averages were obtained by averaging data from all the available technique, which, in most cases, provided a consolidated result of a given primary NP size.

The six different biophysical techniques used in this study (DLS, TEM, SEM, SAXS, AFM, and wSTEM) provide similar results when characterizing the size of monodispersed nanoparticles (average amplitude variations of 25%). Interestingly, on these monodispersed NPs, the variation in size within each technique (using different experimental setup) is about the same as the variation among all six techniques. A summary of “realistic” NPs sizes determined in this study is found in Table 1. As expected, the average inter-technique deviations increased when size characterization involved agglomerated NPs or multimodal distributions; a maximum amplitude variation of 300% was found between DLS and EM. Most importantly, variations in size determination was not only an intrinsic limitation of each individual technique but included: (1) the presence of mixed size NPs and the lack of recognition of some minor populations; (2) the degradation of NPs; (3) the operational aspects (experimental details that bias unintentionally measurements by capturing/excluding some NPs populations); and (4) the NP agglomeration that challenges the operator for individual NP size measurements (identification of the “correct” edge of NPs for instance). We would like to emphasize that NP size characterization is a complex problem that requires a full attention (especially to details) and cannot be assigned to inexperienced operators/analysts in NP size characterization. We strongly advise combining several biophysical techniques, both direct and indirect, and to perform variations in measurement setups (sample dilutions, various grids for EM, various substrates or imaging modes for AFM, different dispersion protocols for DLS, etc.) and to take the global average of all these techniques as the average size of a NP. For complex NP size distribution (multimodal), a histogram of the distribution is necessary or a report of the different detected modes.

Regarding the applicability of tested techniques on NPs size determination, the two tested indirect techniques, SAXS and DLS, were totally reliable on monodisperse NPs in solutions, whereas only the SAXS technique was reliable for “realistic” heterogeneous NPs. Even with modern analysis in DLS, this technique is of little help to identify primary particles when NPs are agglomerated, but, due to its ease of use, DLS could be a first choice technique for an initial characterization of unknown NP samples, on the condition that the presence of agglomerated NPs be detected by the instrument analysis software. Single NP imaging remains a central method for all kinds of NPs where TEM will be preferred over SEM for smaller NPs, AFM will be preferred for non-spherical NPs or imaging in liquid environment as does the wet-STEM technique. All the direct techniques suffer from a major inconvenience, i.e., the necessity to deposit NPs on a flat surface, which has been shown to cause major operational limitations. The main challenges with EM-based techniques are the precise identification of the edge of NPs to provide accurate size values and the lack of characterization in third dimensions could be a major hurdle for non-spherical NPs. Wet-STEM is a relatively new EM-based technique for NPs; it allows the imaging of NPs while in liquid solution and may become a critical advantage when NP agglomeration is provoked through drying. AFM technique is another direct technique, but, to the contrary of EM-based techniques, AFM images contain physical height measurements and can provide size values in three-dimension (with an x–y plane limitation due to tip convolution). Thus, AFM is likely the method of choice when the shape of NPs is far away from spherical. Evaluating the shape of NPs remains the next important challenge in nanotoxicology.

## Figures and Tables

**Table 1:** Global average values for NP size determination.

Family	NPs	Direct Methods			All Methods
		All Data Pooled (Mean ± SD in nm)	*n*	Technique Average (Mean ± SEM in nm)	Technique Average (Mean ± SEM in nm)
Silver	NM-300K	16.8 ± 12.0	2588	18.6 ± 3.8 (*n* = 6)	17.1 ± 3.5 (*n* = 8)
	PVP	53.9 ± 18.3	960	53.3 ± 3.1 (*n* = 6)	55.8 ± 3.7 (*n* = 7)
TiO_2_	A12	10.5 ± 3.8	1269	13.6 ± 2.1 (*n* = 6)	22.5 ± 8.4 (*n* = 8)
	P25	21.3 ± 10.0	2220	21.5 ± 1.5 (*n* = 6)	23.5 ± 2.3 (*n* = 7)
	E171A	94.8 ± 59.5	1171	95.2 ± 12.9 (*n* = 6)	101.3 ± 12.5 (*n* = 7)

## Supplementary Materials

The following are available online at http://www.mdpi.com/2079-4991/9/1/18/s1, Content of Supplementary Data: Text: Difficulties and recommendations for evaluating NPs size, Table S1: Global results of silver and TiO_2_ nanoparticle size measurements provided by all the participants, Figure S1: Wet-STEM time series for silver NM-300K nanoparticles, Figure S2: High magnification of NM-300K NPs observed with wSTEM. Figure S3: Wet-STEM time series for PVP-coated silver nanoparticles, Figure S4: TEM images of silver NM-300K NPs on two different grid supports, Figure S5: Two different methods to measure NPs sizes (Ludox TM-50).
