# Supplementary material for: On the Operational Aspects of Measuring Nanoparticle Sizes
Source: Nanomaterials (Basel). 2018 Dec 23;9(1):18. doi: 10.3390/nano9010018 (PMC6359205; doi:10.3390/nano9010018)
Supplement: Supplementary file 1 [file nanomaterials-09-00018-s001.pdf]

nanomaterials

Article

On the Operational Aspects of Measuring Nanoparticle Sizes

Jean-Marie Teulon [1,2,†], Christian Godon [2,3,†], Louis Chantalat [2], Christine Moriscot [1], Julien Cambedouzou [4,‡], Michael Odorico [2,4], Johann Ravaux [4], Renaud Podor [4], Adèle Gerdil [5], Aurélie Habert [5], Nathalie Herlin-Boime [5], Shu-wen W. Chen [6], Jean-Luc Pellequer [1,2,*]

*To whom correspondence should be addressed: jean-luc.pellequer@ibs.fr

Content of supplementary data:

Text: Difficulties and recommendations for evaluating NPs size

Table S1: Global results of silver and TiO_2 nanoparticle size measurements provided by all the participants

Figure S1: Wet-STEM time series for silver NM-300K nanoparticles

Figure S2: High magnification of NM-300K NPs observed with wSTEM

Figure S3: Wet-STEM time series for PVP-coated silver nanoparticles

Figure S4: TEM images of silver NM-300K NPs on two different grid supports

Figure S5: Two different methods to measure NPs sizes (Ludox TM-50)

The following paragraphs describe in detail the difficulties encountered for determining the size of nanoparticles as well as recommendations on how to optimize this process.

Difficulties in evaluating the NP size with AFM imaging

To evaluate the width of a single object deposited on a flat substrate using AFM requires a minimum sampling resolution, i.e. size of recorded pixels in nm. At optimal imaging resolution (1 x 1 μm² and 1024 pixels²) the sampling resolution is of 0.97 nm / px (or ~ 1 nm / px). Consequently, at such resolution, the precision of measurement is about 0.5 nm (1/2 of sampling resolution). Unfortunately, this would be true if the AFM tip (surface probe) was infinitely narrow. Currently, commercial cantilevers are provided with a 1 or 2 nm tip radius, at best. Thus, AFM images do not reproduce true shape of objects but rather a convolution of their size with that of the imaging cantilever. Several treatments of tip convolution effect have been devised [1-3] but all require a good knowledge of the geometry of the tip and successful reconstruction is rare on biological objects. Nevertheless, the tip convolution problem in AFM is only present in the lateral dimension of objects and thus does not occur when measuring their maximum height. Accordingly, height measurements in AFM are much more accurate than lateral dimensions. In addition, the precision of instrumental height recording is given by its 16-bit digital converter of the maximum z-piezo range (5.32 μm in our case); thus the precision in height recording in contact mode is about 5.32 / 65536 = 0.8 Å. Thus, when imaging spherical NP size with AFM, height values are true diameter equivalent.

The main difficulty in imaging NPs with AFM is the presence of agglomerates of very different sizes. Large change in object sizes makes the AFM regulation challenging in all kinds of oscillating imaging modes. The major risk is the contamination/damaging of the AFM tip which will invariably end up in poor image qualities. Such difficulties have been encountered in this work with all TiO_2 NPs: A12, P25 and E171, a simple consequence of the agglomerated state of these NPs. An effective workaround is to reduce the imaging speed to about 0.25 Hz per line which makes the capture of less than an image per hour with an additional risk of drifts. However, despite the fact that some

images are distorted in some parts of the image, other parts could be analyzed correctly (unless the tip has been irremediably damaged).

The critical limitation in height measurements with AFM is the definition of the background floor. Due to the nature of the piezoelectric tubes used in AFMs and the substrate (mica) orientation, AFM images are not parallel to a "virtual ground floor". The process that corrects image tilt and image roughness are common processes in AFM but can be particularly tricky when the "background floor" of the image is hidden by particles. Correction for such problems is an iterative process that requires to perform preliminary "flattening" of AFM images followed by additional "flattening" operations excluding as many particles as possible. Best results are only obtained when a significant surface of the background has no NPs.

Good practice in NP size determination using AFM

Similarly to other microscopy techniques, AFM requires to deposit samples on a flat substrate. As seen for electron microscopy, artifacts do happen because deposited particles need to have a significant interaction with the flat substrate; otherwise some population of particles may not be observed due to their elimination at the rinsing or drying stages. Besides, it has been shown that the commonly used mica surfaces are prone to artifacts by contributing to the destabilization of fragile NPs such as silver NPs (Fig. 5). Consequently, it is important to test several imaging conditions by varying for instance the chemical nature of the flat substrate [4] or the concentration of deposited particles. Indeed, care should be taken to avoid depositing highly concentrated NPs which may reduce the efficiency of height measurements due to NPs agglomeration. It has been discussed above that a critical step in AFM image is to obtain a flat background. Consequently, it is critical to anticipate AFM scanning by allowing enough space around particles so that an efficient flattening procedure can restore a perfect background. If the scan size only shows agglomerated NPs, there is currently no practical methods to flatten such images.

Equally important is the number of measurements to be performed when measuring NP sizes at single particle level. It is important to collect enough measurement on isolated elements in order to characterize the distribution of the entire population. Obviously, this cannot be made with only a dozen size measurements. From our experience, we have shown that a minimum of 100 measurements is necessary with a better target around 300 measures; but going from 300 to 1000 measurements did not change significantly the shape of the population distribution. Thus, we anticipate that an appropriate number of measurements be located between 100 and 300 if there is a significant variation in individual NP size. It is, however, acknowledged that such a number could be difficult to obtain for a reasonable resource usage (as found in a couple of measurements of this study).

There are two main approaches to obtain NP height values from AFM images: using a cross-section profile (mostly used in this work, **Fig. S5**) or selecting individual particles using a threshold. The former is less sensitive (but not exempt) to the "flatness" of the AFM image than the latter approach. For more accurate results, cross-sections must be performed horizontally along the AFM fast-scanning line. A cross-section may have a user-defined thickness in which the analysis program is averaging values below the cross-section; according to our experience, thickness should be at most ¼ of the size of the object (in pixel units). The height of a NP using the cross-section approach is determined by subtracting two thresholds: one for the background floor and one for the maximum height of the NP in the AFM image. This approach is strictly manual and thus tedious to repeat several hundred times. It is, however, very helpful when NPs are highly agglomerated or where only NPs at the border of the agglomerate can be safely measured. The second approach to obtain NP height values is the automated selection of particles, often called "grains" in analysis software. The principle is to use a threshold value above which any pixel of objects on AFM images is considered as a NP. When the grains are selected, and on the condition that they are well isolated from each other, it is easy to detect the pixel having the maximum height within these grains. This approach provides statistically relevant information (as many as there is individual NP in one AFM image) but is strongly dependent upon perfect AFM image flattening. Indeed, for agglomerated or

aggregated NPs, this approach is quite challenging and should only be used with great care. Similarly to the first approach, the height of a NP is obtained by subtracting the height of the background floor to that of maximum NP height values determined for each grain. A third approach has also been used in this work. If the AFM image contains accolated NPs, then horizontal cross-section lines could be drawn over tightly packed NPs. By measuring the total length from apex to apex and divide by the number of NPs, it is possible to use lateral dimensions with AFM imaging without encountering the problem of tip convolution (remember that there is no tip convolution effect at the apex of particles). Sometimes, NPs are not aligned horizontally and then the cross-section has to be performed in the appropriate direction, at the cost of possible errors in pixel alignments.

Difficulties in evaluating the NP size with TEM imaging

Although sample preparation for Transmission Electron Microscopy does not require really specific skills, the main difficulty in the TEM technique remains the behavior of the sample once adsorbed on the grid. Deposited particles need to have a significant interaction with the support film. This is of particular importance with the carbon-floatation technique in which the particles may distribute differently between the two surfaces (mica and carbon). In addition, weakly bound NPs may also fall into the well during the floatation step. Direct deposition of NPs on carbon grid avoids the partition among two surfaces but still suffers displacement (or elimination) during the blotting step. Another drawback due to sample deposition is the possibility of flattening effect that can occur during drying. In some cases, we have to glow discharge the carbon-coated grid to change the hydrophobicity of the carbon (negative or positive glow discharge). Obviously electron microscopes are quite expensive instruments with usually a high yearly maintenance cost and require accessory instruments such as carbon coating and glow discharge devices. To reduce the cost, EM is often available in core facilities and sometimes in open access. It is therefore a necessity to discuss with an experienced operator about the property of your sample.

TEM instruments produce 16-bit gray images of a sample and determining the precise size of NPs is challenging. There are several possibilities to obtain NP sizes with TEM data. If NPs are well isolated, it is possible to use image processing software (like ImageJ, [5]) to select well-contrasted NPs and to measure a pixel-based surface area (or similar parameters such as Feret's diameter) which in turn could be translated into diameter with some sphericality approximation. Unfortunately, NPs are rarely isolated on EM grid, either due to natural or artificial agglomerations (drying effects). Consequently, most NP size measurements with TEM images are done manually. Size values could be obtained by manually labeling a given NP using a circle and then deduce the diameter (again, assuming that the NP is spherical), by manually drawing a line from one edge to another edge of a given NP and then measure the length of the line after appropriate software calibration, or by manually drawing a cross-section in the image and then measure the distance between the dark/bright edges of NPs (using Gwyddion for instance). All these methods work relatively well on isolated NPs where clear edges are visible, but it becomes very challenging when NPs are agglomerated like all the TiO_2 NPs in this work.

Good practice in NP size determination using TEM

The grid preparation is a rather straightforward step. Proper sample deposition greatly improves the analysis of the NPs size. Two different ways to deposit the sample on the grid are commonly used and it is strongly suggested from this work to systematically use both of them (see methods). Usually, adsorption of molecules is strong enough so that they are not removed by subsequent rinsing and staining operations which eliminate most of the buffer salts when present. However, since NPs could have different affinities for the carbon film (or the mica), some adjustments such as sample concentration or adsorption time should be explored. Moreover, it has been shown in this study that the type of EM grid impacts the fixation of different NPs, thus it is also suggested to test different types of carbon-coated grids. For most NPs, there is no need to use heavy metal staining since NPs are easily visible on a TEM grid when scanning even at low magnification.

Images at higher magnification are, of course, necessary to precisely measure the NPs size. It is usually possible to obtain enough particles on a single grid by collecting images in several regions. One grid with dried nanoparticles can stay several hours in the microscope and if necessary it is possible to acquire more than hundred images per grid. Magnification can be varied from x22 to x68,000 which translates into a pixel resolution of 0.298 to 0.09 nm, respectively. Accurate measurements imply good quality images (without drift or astigmatism) and a proper calibration of the electron microscope. There are different ways to carry out calibration: mix the sample with a standard or calibrate the microscope before use with calibration tools. Microscope calibration must be performed at least once a year, during maintenance for instance. In some cases, TEM images show a tilt which cannot be easily recovered from classical image processing tools (like ImageJ). It is therefore strongly suggested to same TEM images in native format (.dm3 in our instrument) which can be further open in microscopy analysis software. Gwyddion is able to read several native TEM image formats and to perform appropriate flattening without altering the quality of the image. In case the native file format of the microscope cannot be read in analysis software, it is important to save EM images in TIFF only. The cross-section profile is the suggested method to measure NPs sizes as it performs equally well on isolated or agglomerated particles. For the latter ones, it is suggested to only measure those NPs present at the edge of the agglomerate so that it is possible to clearly distinguish most of the edge of a given NP. A very detailed analysis of uncertainties in NP sizes determination using TEM can be found elsewhere [6].

Difficulties in observing NPs with Wet-STEM imaging

One of the main challenges to perform wet-STEM measurements is to control the concentration of NPs in the deposited sample. It is necessary to dilute the solution prior to its introduction in the ESEM chamber because the concentration of NPs in the sample will increase during the pumping sequence and the thinning of the liquid layer necessary to reach electron transparency. This sample preparation step can strongly modify the properties of the liquid that contains the NPs and it may force agglomeration or dispersion of the NPs in the liquid before its observation. This difficulty arises because the ESEM chamber does not allow a precise control of the water thickness and consequently the final NPs concentration in the liquid layer. The risk being that the aggregation of NPs in the liquid could not really be a representative of the state of the stock solution. Another limit of the use of Wet-STEM is the difficulty to maintain the liquid thin film stable for a long-time experiment. When the thin liquid layer is obtained, most of the observations must be performed within 15 minutes to be sure to maintain the stability of the liquid layer. Furthermore, one can dry the sample completely during the initial pumping sequence and later condense water on the dried NPs. If this occurs, there is no real possibility to check if this has occurred as it is not possible to use the electron imaging during this sequence. One proof for the sample not to have been dried during the pumping sequence is to observe the motion of NPs in the liquid layer during its characterization. Indeed, if the sample has been dried, the NPs bind to the carbon film of the TEM grid and even if water is condensed on these NPs, they remain stuck on the carbon layer, and they do not move during their observation.

Image contrast can also vary depending on the thickness of the water layer as reported recently [7]. Even if this effect has not been directly observed in the present study, these authors report contrast inversion between the water layer and gold NPs as a function of the thickness of the water layer. In some particular conditions, gold NPs cannot be distinguished from the water layer.

Another difficulty directly linked with the wet-STEM technique lies in the fact that the electron beam modifies locally the chemistry of water due to the irradiation effects. This phenomenon is well-known and reported for the observation of liquids by TEM in dedicated closed cells [8]. These local modifications can yield to unexpected attraction – and agglomeration - or repulsion of the NPs in/out of the zone of observation. Finally, if surfactants (that are generally organic molecules) are present in the liquid, they can be degraded due to the effect of the electron beam and the degradation products can limit or prevent from observing the NPs. The effect of the electron beam can also yield to a local increase of the liquid temperature and finally to the drying of the solution.

Depending on the nature of the NPs, and particularly if they contain organic matter, they can possibly be degraded by the electron beam. This was the case with the PS22 NPs that was sensitive to the electron beam.

The fastest recording rate that can be achieved using the wet-STEM mode is 20 images per second. But in these conditions, the image quality is relatively poor. Thus, to ensure the formation of an average quality image, 1 image per second is a good compromise. However, it is sometimes impossible to observe directly isolated NPs as their movements due to the Brownian motion is too fast. In this case, they appear as a small strike on the images and it is not possible to characterize their size and numbers. In addition, some NPs stick by capillary forces to the carbon support layer and thus the NPs are not in their initial state (i.e. free of movement in the liquid) and what is observed is not really the NPs in the liquid.

Good practice in NP size determination using wet-STEM

The wet-STEM stage is easy to implement in any Environmental SEM (ESEM). Although the thinning of the liquid film can be tricky during introduction of the sample in the ESEM chamber, it takes no more than 30 minutes from the grid preparation to sample observation. When the water vapor in the ESEM chamber is properly controlled, a sample can be observed up to 4 hours which provides enough time for probing several positions and obtaining the distribution of the NPs in the solution and to record a sufficient number of images. The continuous recording of images on the same zone of the sample with a fast scan rate allows a precise description of the motion of the NPs, at the 10-100 nm level. It also allows the visualization of the NPs aggregation routes in liquid and describes the degree of dispersion of the NP in the solution (as the sample is never dried). Furthermore, the degrees of freedom of the agglomerated NPs can be determined by looking at their relative rotations within the aggregates. Even if the mobility of NPs is high during image recording, it is often possible to observe isolated NPs when they bind to the carbon film of the TEM grid. It is thus also important to scan for carbon areas of the TEM grid. Finally, when the liquid film totally evaporates, it is a good practice to continue to record images to perform a "dry" analysis of NP sizes.

Analysis of the NP size can be achieved precisely by using the image of isolated NPs that stick on the carbon coating. The precision on the individual size determination is directly linked to the thickness of the water layer that is surrounding the NP. Indeed, the primary electron beam is scattered by the water layer, and the thicker the water layer, the larger the scattering effect. The scattering of the electron beam results as a blurring of the NP image leading to an enlargement of its apparent diameter; the smaller the NP the higher the error on its diameter. Thus, when the water layer is thick, the smallest NPs cannot be observed directly in liquid (case of the silica particles dispersed in the SM30 sample). This technique is particularly well adapted for the observation of NP diameters ranging from 10 to 200 nm when dispersed in aqueous liquids (sometimes up to 1000 nm depending on their electronic contrast and density). However, changes in image contrast and scattering effects can sometimes generate difficulties in the interpretation of the recorded images [7]. Finally, different liquids than water can also be used if they are compatible with the pressure domain allowed by the ESEM pumping system (10-2400 Pa) and if they are compatible with the pumping system of the microscope [9]. Condensation of pure liquid ethanol has been achieved successfully in the ESEM chamber in our laboratory (unpublished data). Finally, due to the scanning procedure used to obtain wet-STEM images, it is more accurate to measure distances with a horizontal line (as opposed to vertical or oblique lines), similarly to what is suggested for AFM.

Difficulties in evaluating the NP size with SAXS

SAXS formalism theoretically allows a quantitative determination of the shape, size and concentration of particles of nearly spherical symmetry provided their size does not exceed the maximal value corresponding to the minimal accessible scattering angle. However, in the case of nanoparticles under study in this work, several difficulties have to be taken into account.

First, particles should remain in a dispersed regime, i.e. they should be in sufficiently low concentration in order not to interact. Too high concentration would indeed result in a so-called

"structure factor" originating from interferences between regularly organized particles. This structure factor would modify the scattered intensity and hinder a part of the "form factor" characteristic of the particle's shape and size. However, too dilute a suspension would also result in too low a signal, preventing a correct characterization of the particles. As the SAXS intensity is also related to the electronic contrast between the particles and the surrounding media, the balance can be very difficult to reach. Another difficulty comes up when nanoparticles size distribution becomes too important. SAXS data can be fitted with numerical models of different geometrical shapes, and can account for a size polydispersity described by various distributions (Gaussian, Lorentzian, Voigt,…). Unfortunately, the larger these distributions, the smoother SAXS intensity modulations become, and several hypotheses might result in good qualitative agreement between intensity calculations and the experimental profile. There is therefore a risk of "over-interpretation" of the experimental data.

Good practice in NP size determination using SAXS

In order to face the first difficulty described above, several dilutions should be made for a given nanoparticle sample in order to reach the best compromise between signal to noise ratio (high for high particle concentration) and prevention of nanoparticles aggregation. This can be achieved by verifying that the SAXS profile is not modified upon decrease of the nanoparticle concentration until the loss of reasonably high signal. If a significant change is observed in the SAXS profile while decreasing the concentration, this could testify to nanoparticle interaction at the higher concentrations. Therefore, SAXS data originating from lower concentrations should be considered for the size and shape analysis. Concerning the risk of over-fitting experimental data leading to misleading conclusions, the use of fitting parameters should always be kept as low as possible in order to be compatible with the experimental profile. This is a general recommendation made for any fitting procedure of experimental data of any origin. Moreover, in the case of SAXS, scattering profiles may appear very smooth, without any remarkable feature, on which first fitting parameters could be estimated. In this case, it is probably safer not to try to go further in the SAXS profile fitting and turn to other characterizations techniques.

Table S1. Global results of silver and TiO_2 nanoparticle size measurements provided by all the participants.

Methods	Parameters	Silver		TiO_2		
		**PVP**	**NM-300K**	**A12 (OcTi 147)**	**P25 (Degussa)**	**E171 (A)**
	Appearance	Agglomerated	Isolated	Agglomerated	Agglomerated	Mostly agglomerated
	Distribution	Monomodal	Monomodal	Monomodal	Multimodal	Multimodal
	Modes (nm)	40-45	10-11	12 nm	13<br>25<br>33<br>61	10<br>35<br>80
AFM	Mean size ± SD (nm)	38.5 ± 12.4	10.4 ± 2.2	11.4 ± 4.1	26.0 ± 13.4	38.4 ± 41.8
	CV (%)	32	21	36	52	109
	Median size (nm)	40.4	10.3	10.7	25.0	19.5
	n	152	690	104	381	183
	Images	4	3	6	37	28

	NPs < 100 nm	**100 %**	**100 %**	**100 %**	**100 %**	**88.5 %**
	Appearance	Isolated	Isolated	Agglomerated	Agglomerated	Agglomerated
	Distribution	Monomodal	Monomodal	Monomodal	~Monomodal	Bimodal
TEM1	Modes (nm)	66-70	17-18	9-10	~21-22	60-70<br>120-130
Grenoble	Mean size ± SD (nm)	58.2 ± 19.8	16.6 ± 3.1	9.4 ± 2.9	20.5 ± 6.2	102.0 ± 39.1
	CV (%)	34	19	31	30	38
	Median size (nm)	59.0	16.7	9.4	20.1	96
	n	475	840	417	547	147
	Images	15	12	11	12	18
	NPs < 100 nm	**99%**	**100%**	**100%**	**100%**	**54 %**
	Appearance	-	-	Agglomerated	Mostly isolated	Agglomerated
	Distribution	-	-	Monomodal	~Monomodal	Multimodal
	Modes (nm)	-	-	9-10	~7-8	10-20<br>60-70<br>90-100
TEM2	Mean size ± SD (nm)	-	-	9.6 ± 2.7	9.8 ± 4.6	38.2 ± 35.7
Grenoble	CV (%)			28	47	93
	Median size (nm)	-	-	9.6	8.5	20.5
	n	-		426	291	70
	Images	-	-	7	22	10
	NPs < 100 nm	-	-	**100 %**	**100 %**	**90 %**
	Appearance	Isolated	Isolated	Isolated	Agglomerated	Agglomerated
	Distribution	Monomodal	Bimodal	Monomodal	Monomodal	Bimodal
	Modes (nm)	50	15<br>57	27.5	18	25<br>135
wet-STEM	Mean size ± SD (nm)	50.5 ± 11.6	34.7 ± 23.1	22.0 ± 5.6	19.0 ± 5.8	120.3 ± 67.9
	CV (%)	23	67	25	31	56
	Median size (nm)	51.4	14.7<br>56.0	21.7	17.9	22.1<br>150.0
	n	114	132<br>123	20	52	56<br>155

	Images	3	14	1	2	4
	NPs < 100 nm	**100%**	**100%**	**100%**	**100 %**	**42 %**
	Appearance	Isolated	Isolated	Agglomerated	Agglomerated	Agglomerated
	Distribution	Monomodal	Bimodal	Monomodal	Monomodal	Monomodal
	Modes (nm)	62	15<br>53	7.5	18	75
STEM	Mean size ± SD (nm)	55.6 ± 16.1	23.5 ± 15.0	9.5 ± 3.4	22.8 ± 8.5	97.2 ± 33.3
	CV (%)	28		36	37	34
(after drying)	Median size (nm)	58.8	15.9<br>48.8	9.4	21.0	91.8
	n	110	255<br>72	68	49	231
	Images	3	2	3	3	3
	NPs < 100 nm	**100%**	**100%**	**100%**	**100%**	**59%**
	Appearance	Agglomerated	Agglomerated	Agglomerated	Agglomerated	Agglomerated
	Distribution	Monomodal	Monomodal	Monomodal	Multimodal	Multimodal
	Modes (nm)	60	13	17	10-12<br>22-24<br>44-46	80<br>140…
SEM	Mean size ± SD (nm)	58.1 ± 14.2	14.6 ± 3.8	18.2 ± 3.8	25.2 ± 9.0	118.8 ± 64.5
Saclay	CV (%)	24	26	21	36	54
	Median size (nm)	58.9	14.3	17.6	23.5	104.1
	n	109	143	55	634	192
	Images[&]	6/15	1/11	1	13	5
	NPs < 100 nm	**100%**	**100%**	**100%**	**100 %**	**47**
	Appearance	Isolated	Agglomerated	Agglomerated	Agglomerated	Agglomerated
	Distribution		Bimodal	Monomodal	Bimodal	Multimodal
	Modes (nm)		3<br>12	11	16<br>24	80<br>130…
TEM	Mean size ± SD (nm)	59 ± 18<br>[10]	11.5 ± 6.9	11.0 ± 2.7	19.5 ± 6.1	115 ± 53
Saclay	CV (%)	31	60	25	31	46
	Median size (nm)		12.0	10.6	18.3	97.5
	n		333	179	266	137

	Images		8		9	10
	NPs < 100 nm		**100%**	**100%**	**100%**	**53%**
BET	Surface specific (m²/g)			82	46	9.4
	density			3.9	3.9	4.0
	Diameter (nm)			19	33	160
SAXS	Mean size ± SD (nm)	ND	ND	12.0 ± 2.1 (only < 30 nm)	ND	ND
	CV (%)			18		
DLS	Distribution	Monomodal	Bimodal	Monomodal		Monomodal
	Mean size ± SD (nm)	71.0 ± 19.9	2.5 ± 0.2<br>22.8 ± 2.2	75.4 ± 0.9	35 ± 5<br>[11]	138.0 ± 7.4
	CV (%)	28	10	1	14	5

AFM: Atomic Force Microscopy, TEM: Transmission Electron Microscopy, wet-STEM: wet Scanning Transmission Electron Microscopy, STEM: Scanning Transmission Electron Microscopy (after sample drying from wet-STEM), SEM: Scanning Electron Microscopy; BET: Brunauer-Emmett-Teller, SAXS: Small-angle Xray Scattering, DLS: Dynamic Light Scattering

n = Number of measures; CV = coefficient of variation (=SD/mean).

& Indicates that only some images out of a given total have been used to perform measurements

ND: Not determine; -: not done

Figure S1

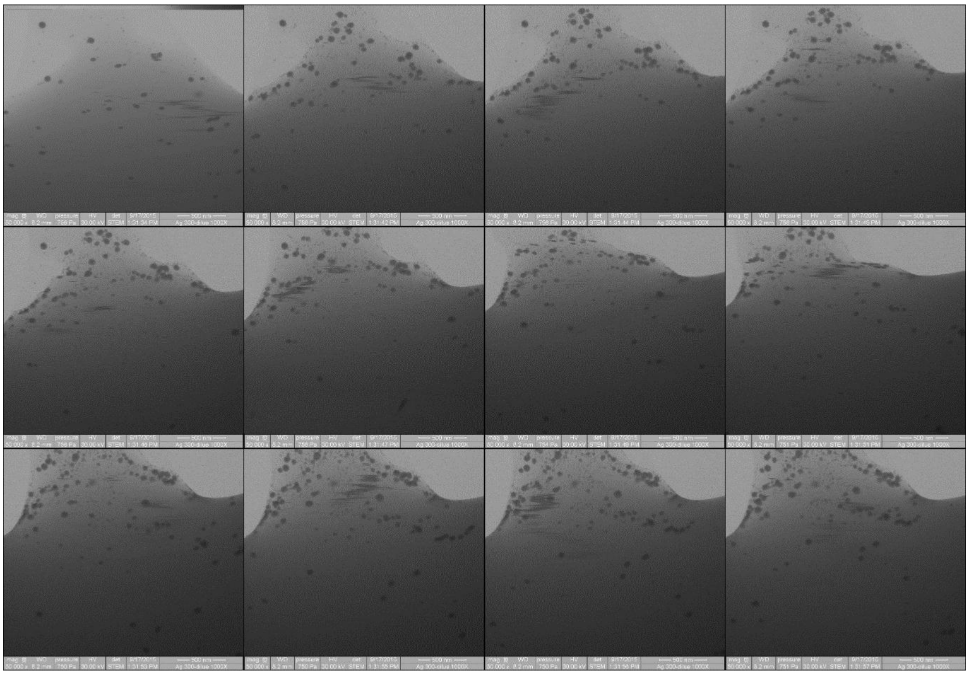

Figure S1. Wet-STEM time series for silver NM-300K nanoparticles. Images were collected about every 1 sec in a liquid environment. Motion of single nanoparticles can be observed from frame to frame. Each image is 2.5 μm² in size. It is interesting to note the absence of an agglomeration of NPs in liquid.

Figure S2

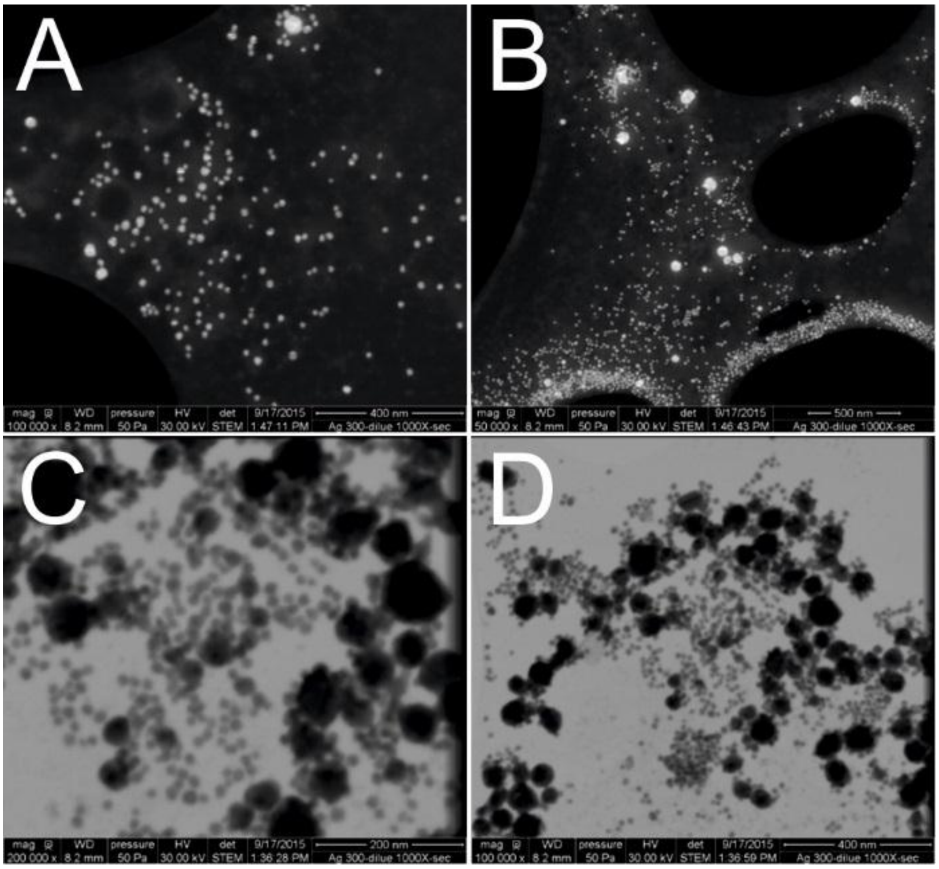

Figure S2. High magnification of NM-300K NPs observed with wet-STEM in liquid (A and C) then dried in STEM (B and D) of identical areas. Dark and bright field images allow the observation of the presence of large NM-300K NPs in both modes of imaging. Image sizes are: A) 1.25 μm², B) 2.5 μm², C) 0.63 μm², D) 1.1 μm². Image A is identical as that in image 5A.

Figure S3

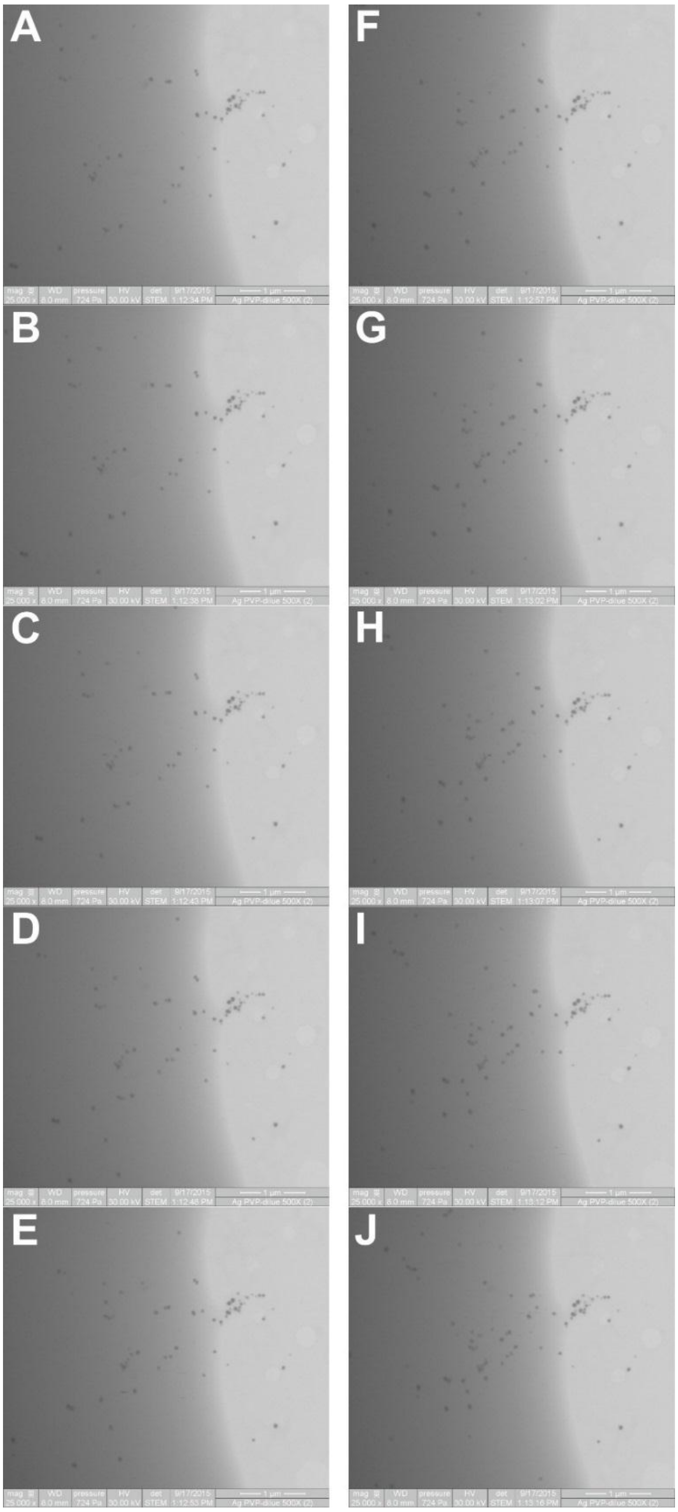

Figure S3. Wet-STEM time series for PVP-coated silver nanoparticles. A-J images were collected about every 5 sec in liquid environment. Displacement of single nanoparticles can be observed from frame to frame. Each image is 5 μm² in size.

Figure S4

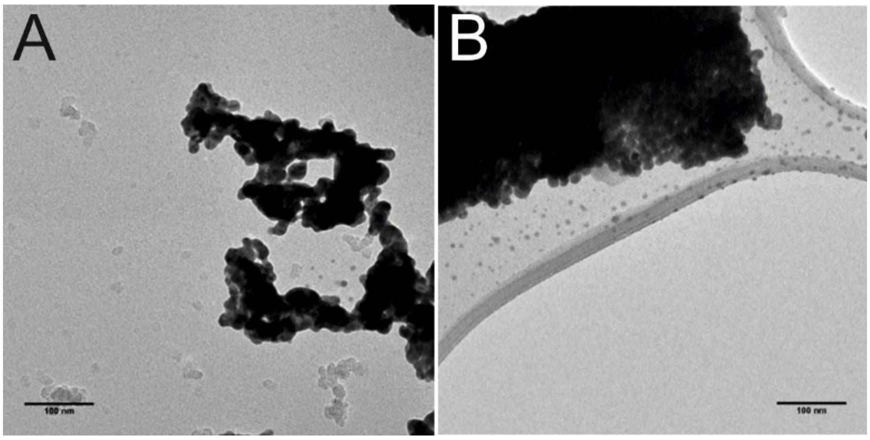

Figure S4. TEM images of silver NM-300K NPs on two different grid supports: A) Carbon film in the absence of holes, and B) Lacey carbon film with the presence of holes in the grid. In absence of holes, mostly large NPs are observed in A) whereas the presence of smaller (light gray) NPs is clearly visible in the carbon grid with holes in B).

Figure S5

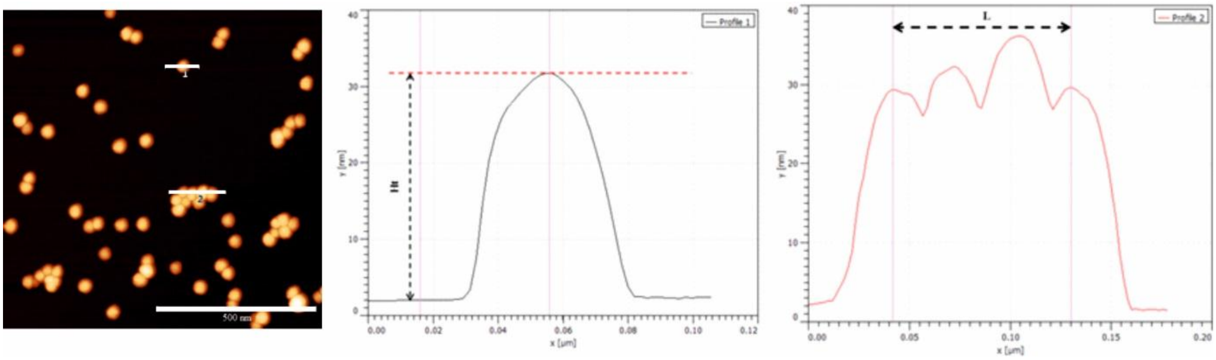

Figure S5. Two different methods to measure NPs sizes (Ludox TM-50). From an AFM image on the left panel, it is possible to draw a cross-section over a single NP in the image using Gwyddion (line 1, middle panel) or over accolated NPs (line 2, right panel). In the first case, the size of NPs is given by a height value which is itself a subtraction of the highest pixels of the NP and those from the bottom background. In the second case, the size of NPs is given by a lateral distance measurement from peak to peak and divided by the number of NPs (three here). Note the convolution effect in AFM measurement in the middle panel where a single NP appears of having a lateral size of more than 40 nm whereas the apparent height is about 30 nm. The scale bar on the AFM image is 500 nm. The color scale is given within the cross-sections. The thickness of cross-section lines has been exaggerated for visibility.

References

1. Villarrubia, J.S. Algorithms for Scanned Probe Microscope Image Simulation, Surface Reconstruction, and Tip Estimation. *J. Res. Natl. Inst. Stand. Technol.* **1997**, *102*, 425–454.
2. Bukharaev, A.A.; Berdunov, N.V.; Ovchinnikov, D.V.; Salikhov, K.M. Three-Dimensional Probe and Surface Reconstruction for Atomic Force Microscopy Using a Deconvolution Algorithm. *Scan. Microscopy* **1998**, *12*, 225–234.
3. Trinh, M.-H.; Odorico, M.; Bellanger, L.; Jacquemond, M.; Parot, P.; Pellequer, J.-L. Tobacco Mosaic Virus as an Afm Tip Calibrator. *J. Mol. Recognit.* **2011**, *24*, 503–510, doi:10.1002/jmr.1118.
4. Godon, C.; Teulon, J.-M.; Odorico, M.; Basset, C.; Meillan, M.; Vellutini, L.; Chen, S.-w.W.; Pellequer, J.-L. Conditions to Minimize Soft Single Biomolecule Deformation When Imaging with Atomic Force Microscopy. *J. Struct. Biol.* **2017**, *197*, 322–329, doi:10.1016/j.jsb.2016.12.011.

5. Schneider, C.A.; Rasband, W.S.; Eliceiri, K.W. NIH Image to Imagej: 25 Years of Image Analysis. *Nat. Methods* **2012**, *9*, 671–675, doi:10.1038/nmeth.2089.
6. de Temmerman, P.; Lammertyn, J.; De Ketelaere, B.; Kestens, V.; Roebben, G.; Verleysen, E.; Mast, J. Measurement Uncertainties of Size, Shape, and Surface Measurements Using Transmission Electron Microscopy of near-Monodisperse, near-Spherical Nanoparticles. *J. Nanopart. Res.* **2013**, *16*, 2177, doi:10.1007/s11051-013-2177-1.
7. Xiao, J.; Foray, G.; Masenelli-Varlot, K. Analysis of Liquid Suspensions Using Scanning Electron Microscopy in Transmission: Estimation of the Water Film Thickness Using Monte-Carlo Simulations. *J. Microsc.* **2018**, *269*, 151–160, doi:10.1111/jmi.12619.
8. Woehl, T.J.; Jungjohann, K.L.; Evans, J.E.; Arslan, I.; Ristenpart, W.D.; Browning, N.D. Experimental Procedures to Mitigate Electron Beam Induced Artifacts During in Situ Fluid Imaging of Nanomaterials. *Ultramicroscopy* **2013**, *127*, 53–63, doi:10.1016/j.ultramic.2012.07.018.
9. Bogner, A.; Jouneau, P.H.; Thollet, G.; Basset, D.; Gauthier, C. A History of Scanning Electron Microscopy Developments: Towards "Wet-Stem" Imaging. *Micron* **2007**, *38*, 390–401, doi:10.1016/j.micron.2006.06.008.
10. Aude-Garcia, C.; Villiers, F.; Collin-Faure, V.; Pernet-Gallay, K.; Jouneau, P.H.; Sorieul, S.; Mure, G.; Gerdil, A.; Herlin-Boime, N.; Carriere, M.; Rabilloud, T. Different in Vitro Exposure Regimens of Murine Primary Macrophages to Silver Nanoparticles Induce Different Fates of Nanoparticles and Different Toxicological and Functional Consequences. *Nanotoxicology* **2016**, *10*, 586–96, doi:10.3109/17435390.2015.1104738.
11. Carriere, M.; Guillard, C.; Pigeot-Remy, S.; Herlin-Boime, N. Impact of Titanium Dioxide Nanoparticle Dispersion State and Dispersion Method on Their Toxicity Towards A549 Lung Cells and Escherichia Coli Bacteria. *J. Translational Toxicol.* **2014**, *1*, 10–20, doi:10.1166/jtt.2014.101.
